# Supplementary figures and images for: POMAShiny: A user-friendly web-based workflow for metabolomics and proteomics data analysis
Source: PLoS Comput Biol. 2021 Jul 1;17(7):e1009148. doi: 10.1371/journal.pcbi.1009148 (PMC8279420; doi:10.1371/journal.pcbi.1009148)

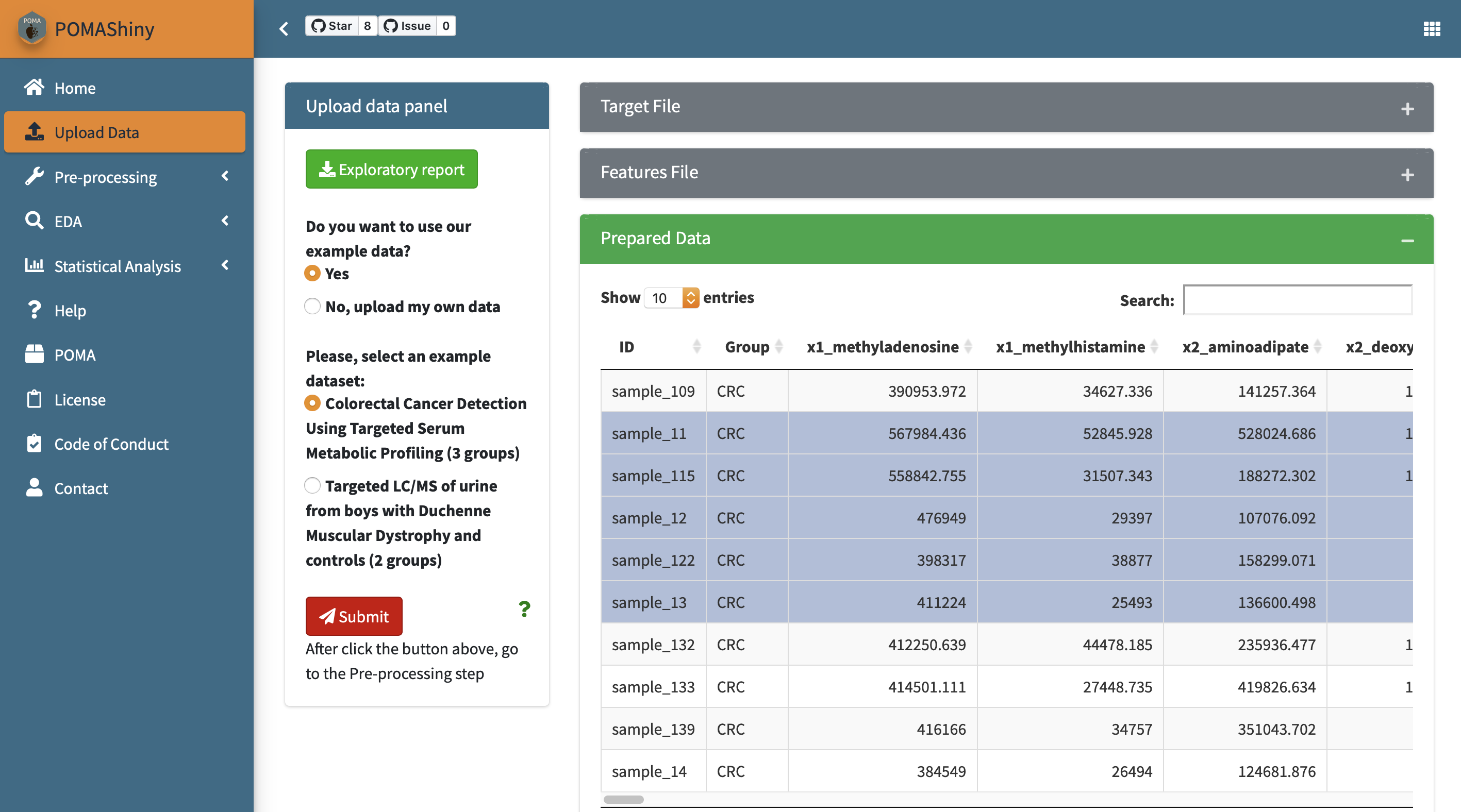

Supplement: S1 Code — In addition to the source code, the archive file contains the documentation for the installation and usage of the app and the Dockerfile to create a Docker image of POMAShiny. (ZIP) [file pcbi.1009148.s001.zip › POMAShiny-1.2.0/inst/png/POMAShiny_demo.png]

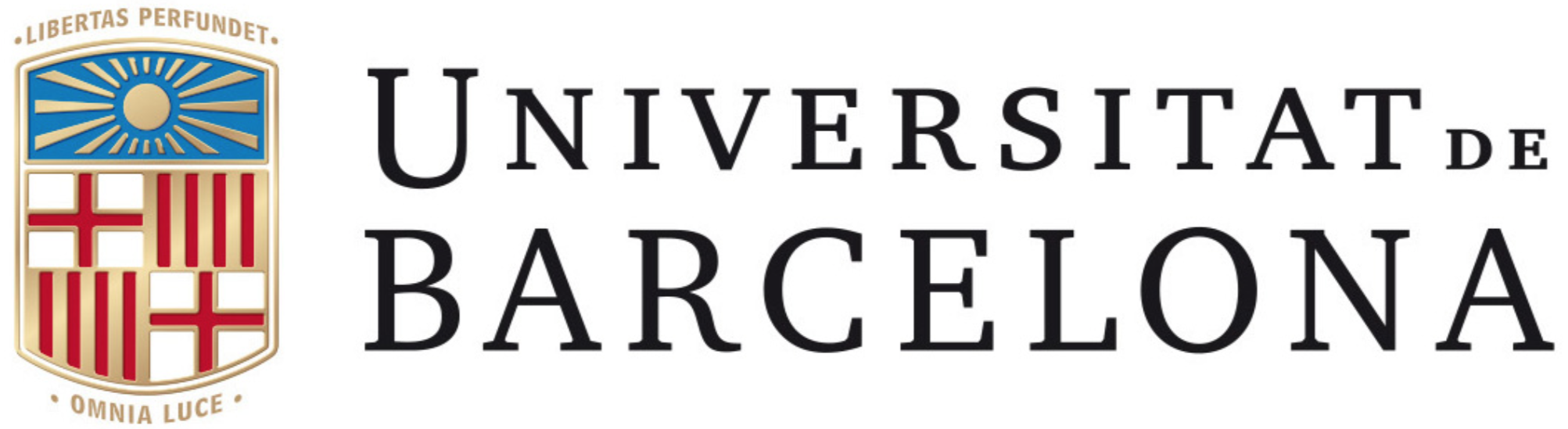

Supplement: S1 Code — In addition to the source code, the archive file contains the documentation for the installation and usage of the app and the Dockerfile to create a Docker image of POMAShiny. (ZIP) [file pcbi.1009148.s001.zip › POMAShiny-1.2.0/app/mds/pix/ub.png]

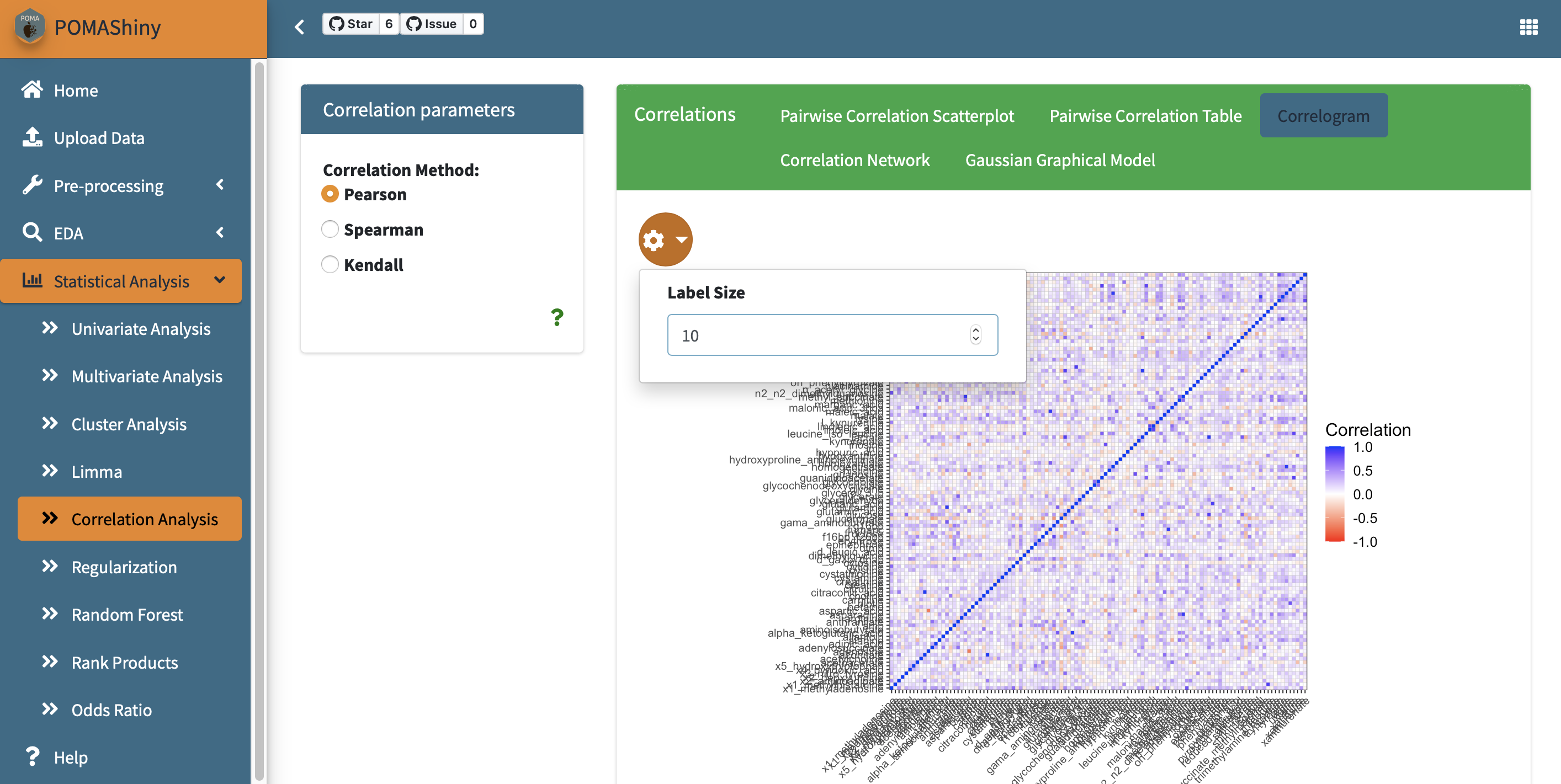

Supplement: S1 Code — In addition to the source code, the archive file contains the documentation for the installation and usage of the app and the Dockerfile to create a Docker image of POMAShiny. (ZIP) [file pcbi.1009148.s001.zip › POMAShiny-1.2.0/app/mds/pix/correlogram.png]

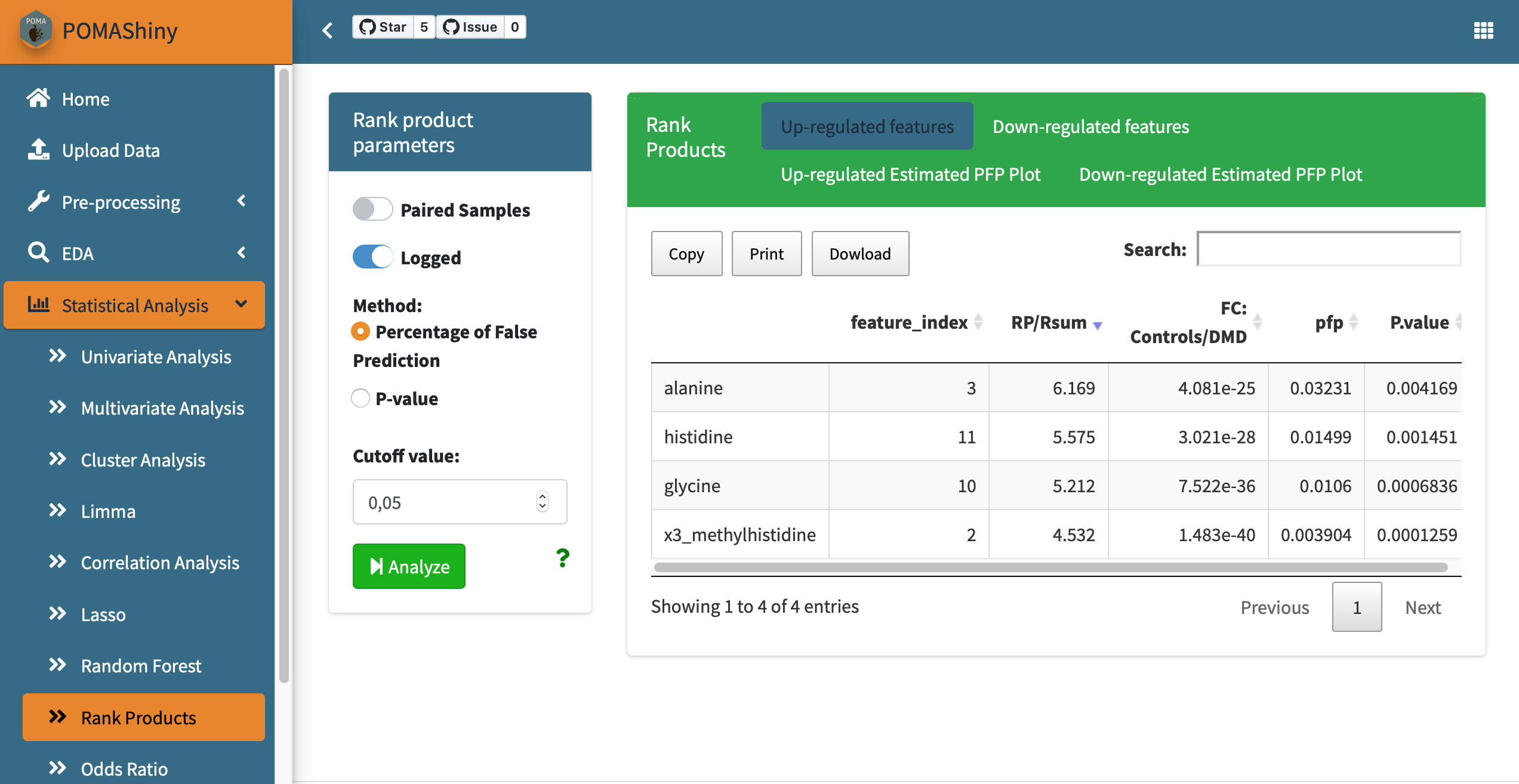

Supplement: S1 Code — In addition to the source code, the archive file contains the documentation for the installation and usage of the app and the Dockerfile to create a Docker image of POMAShiny. (ZIP) [file pcbi.1009148.s001.zip › POMAShiny-1.2.0/app/mds/pix/rank_prod.png]

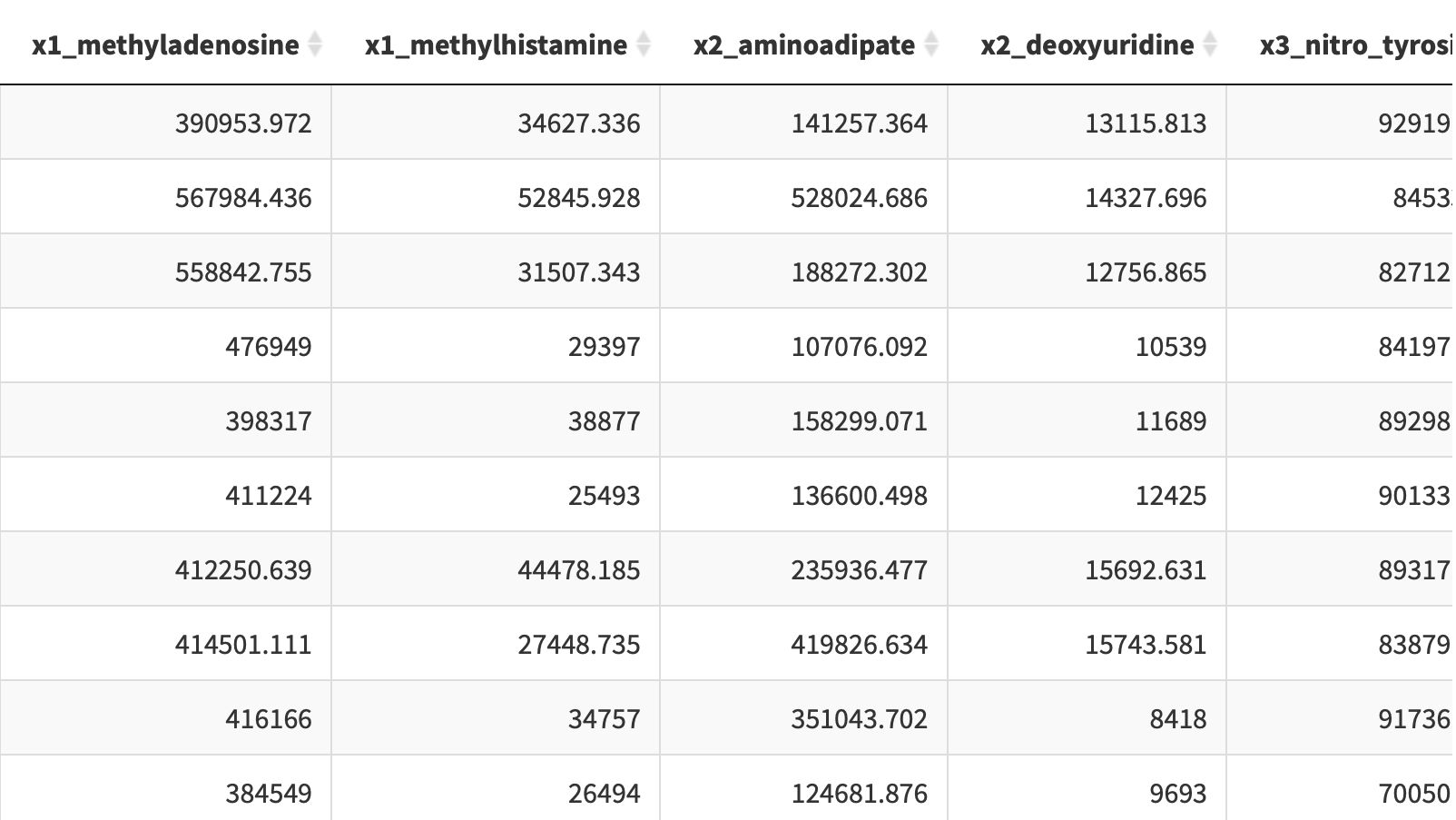

Supplement: S1 Code — In addition to the source code, the archive file contains the documentation for the installation and usage of the app and the Dockerfile to create a Docker image of POMAShiny. (ZIP) [file pcbi.1009148.s001.zip › POMAShiny-1.2.0/app/mds/pix/features.png]

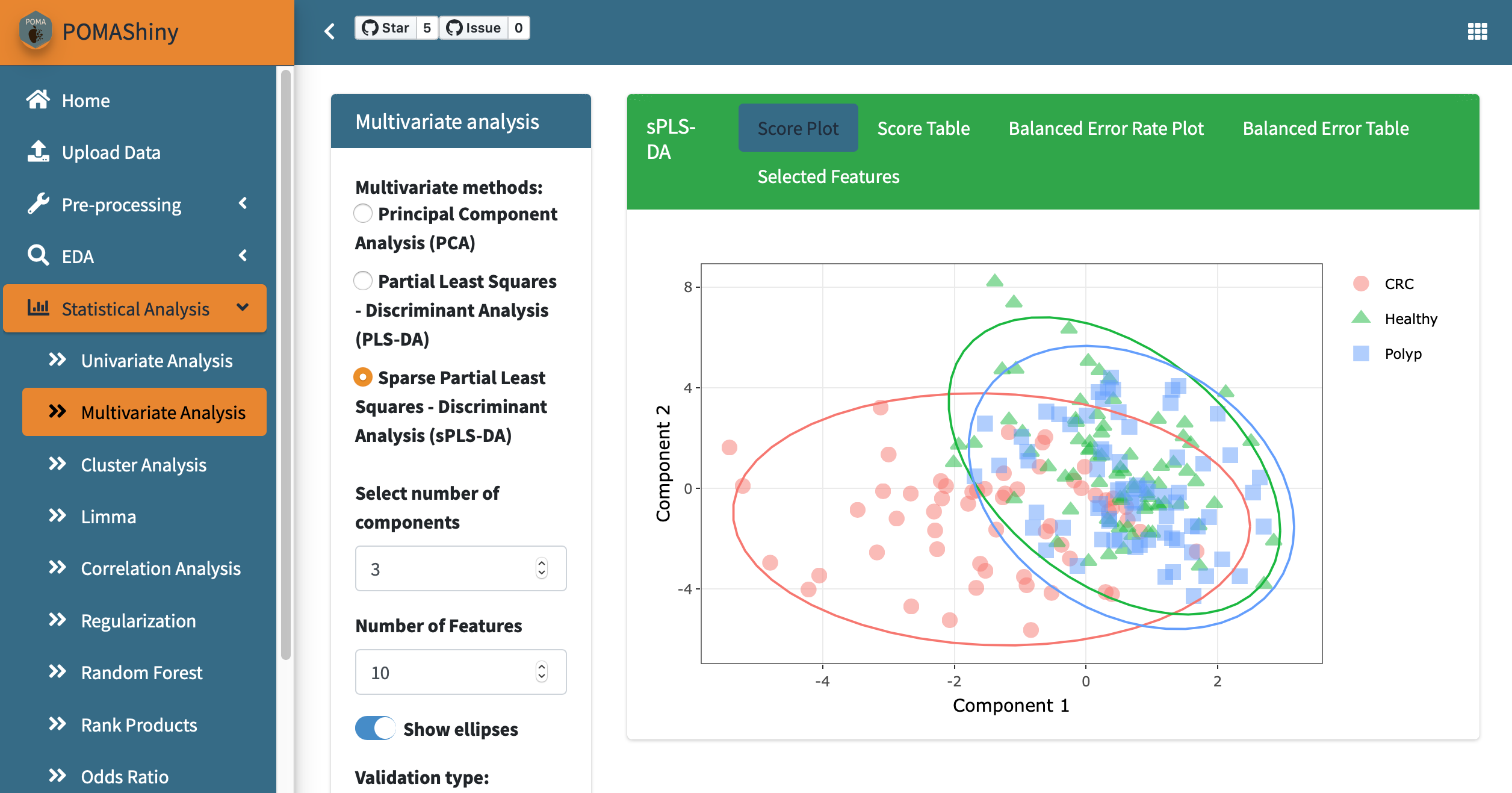

Supplement: S1 Code — In addition to the source code, the archive file contains the documentation for the installation and usage of the app and the Dockerfile to create a Docker image of POMAShiny. (ZIP) [file pcbi.1009148.s001.zip › POMAShiny-1.2.0/app/mds/pix/splsda.png]

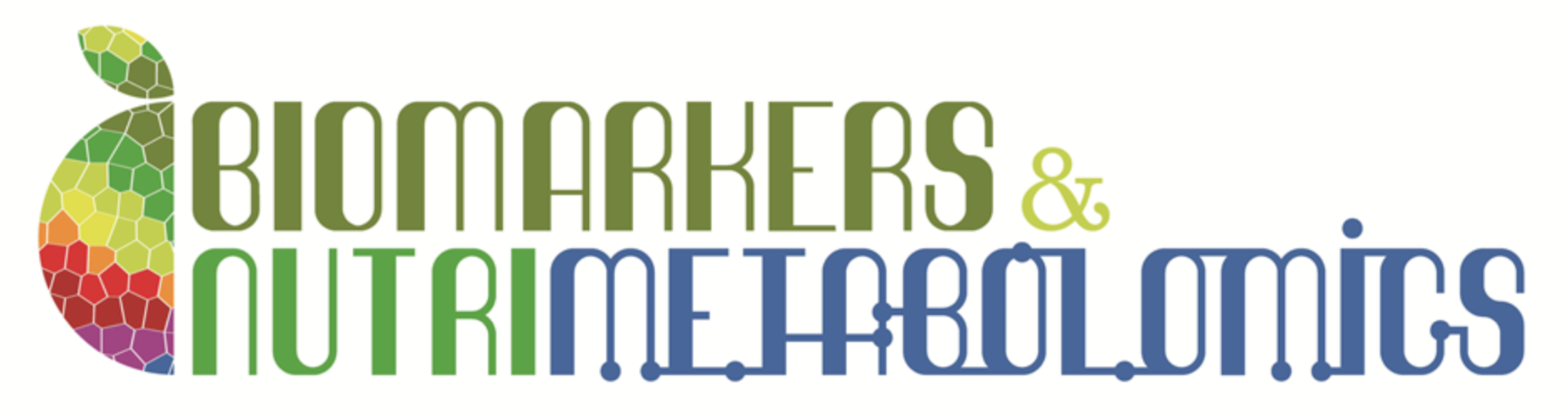

Supplement: S1 Code — In addition to the source code, the archive file contains the documentation for the installation and usage of the app and the Dockerfile to create a Docker image of POMAShiny. (ZIP) [file pcbi.1009148.s001.zip › POMAShiny-1.2.0/app/mds/pix/nutrimetabolomics.png]

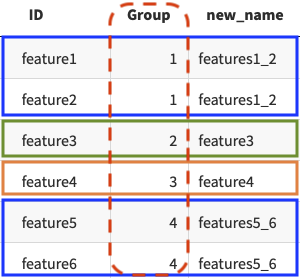

Supplement: S1 Code — In addition to the source code, the archive file contains the documentation for the installation and usage of the app and the Dockerfile to create a Docker image of POMAShiny. (ZIP) [file pcbi.1009148.s001.zip › POMAShiny-1.2.0/app/mds/pix/grouping_file.png]

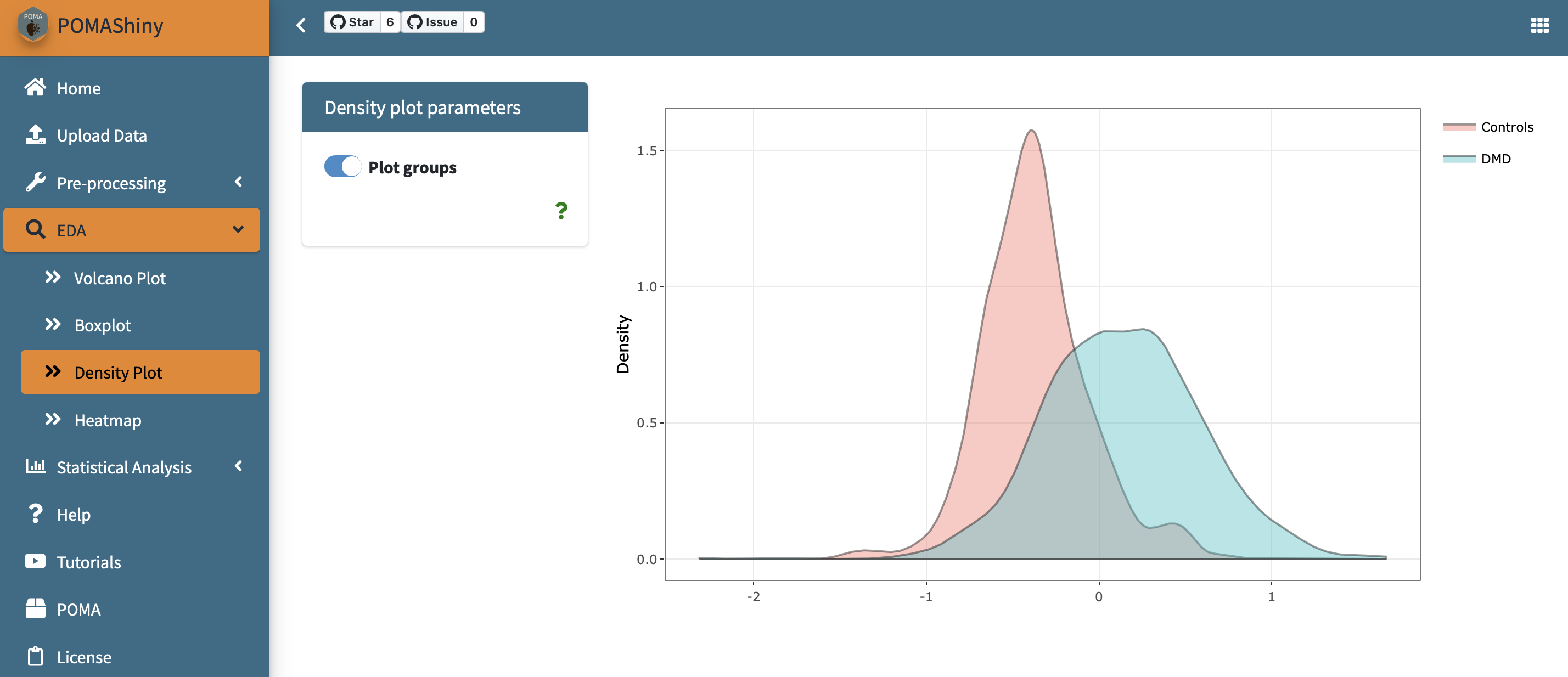

Supplement: S1 Code — In addition to the source code, the archive file contains the documentation for the installation and usage of the app and the Dockerfile to create a Docker image of POMAShiny. (ZIP) [file pcbi.1009148.s001.zip › POMAShiny-1.2.0/app/mds/pix/density.png]

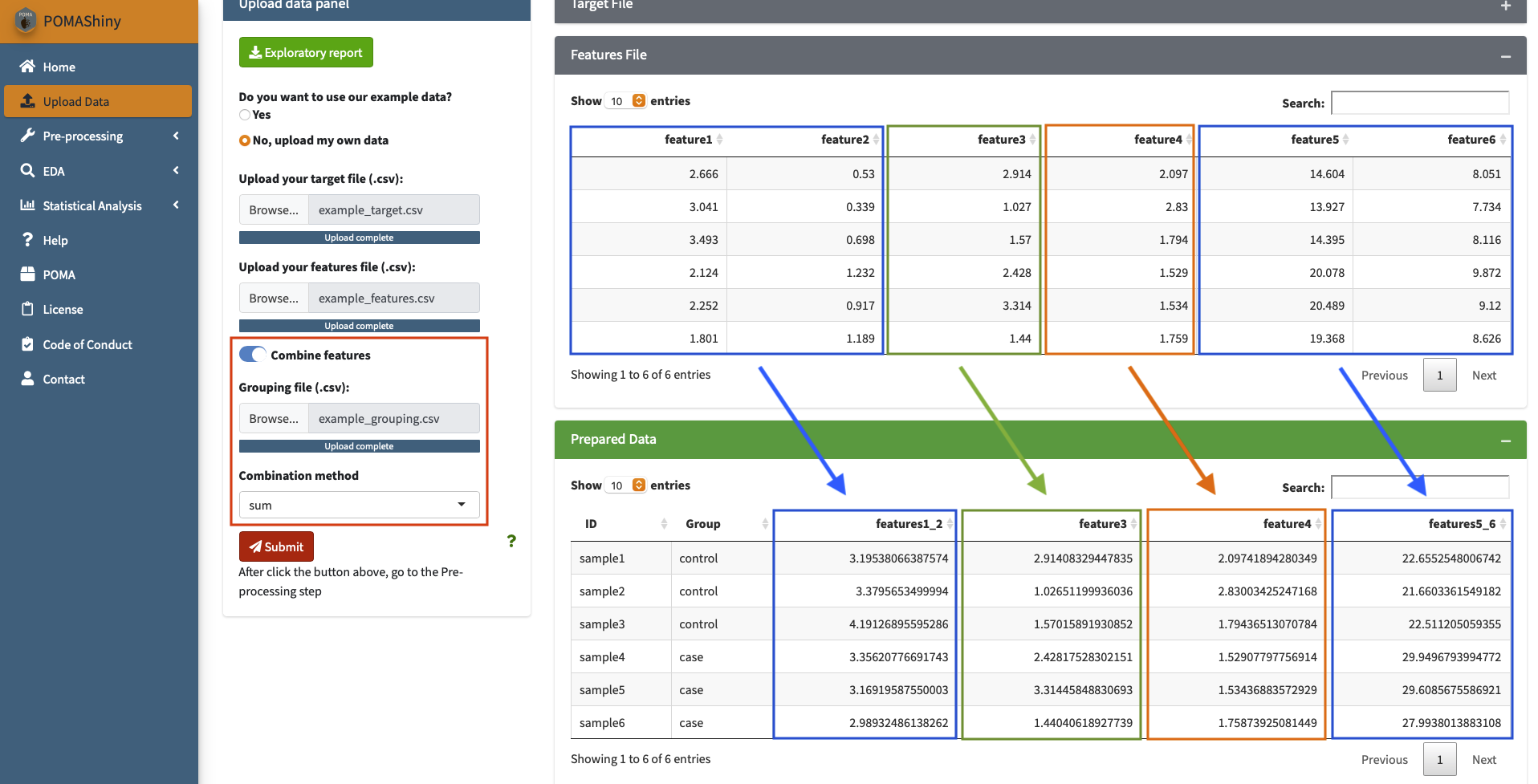

Supplement: S1 Code — In addition to the source code, the archive file contains the documentation for the installation and usage of the app and the Dockerfile to create a Docker image of POMAShiny. (ZIP) [file pcbi.1009148.s001.zip › POMAShiny-1.2.0/app/mds/pix/grouping.png]

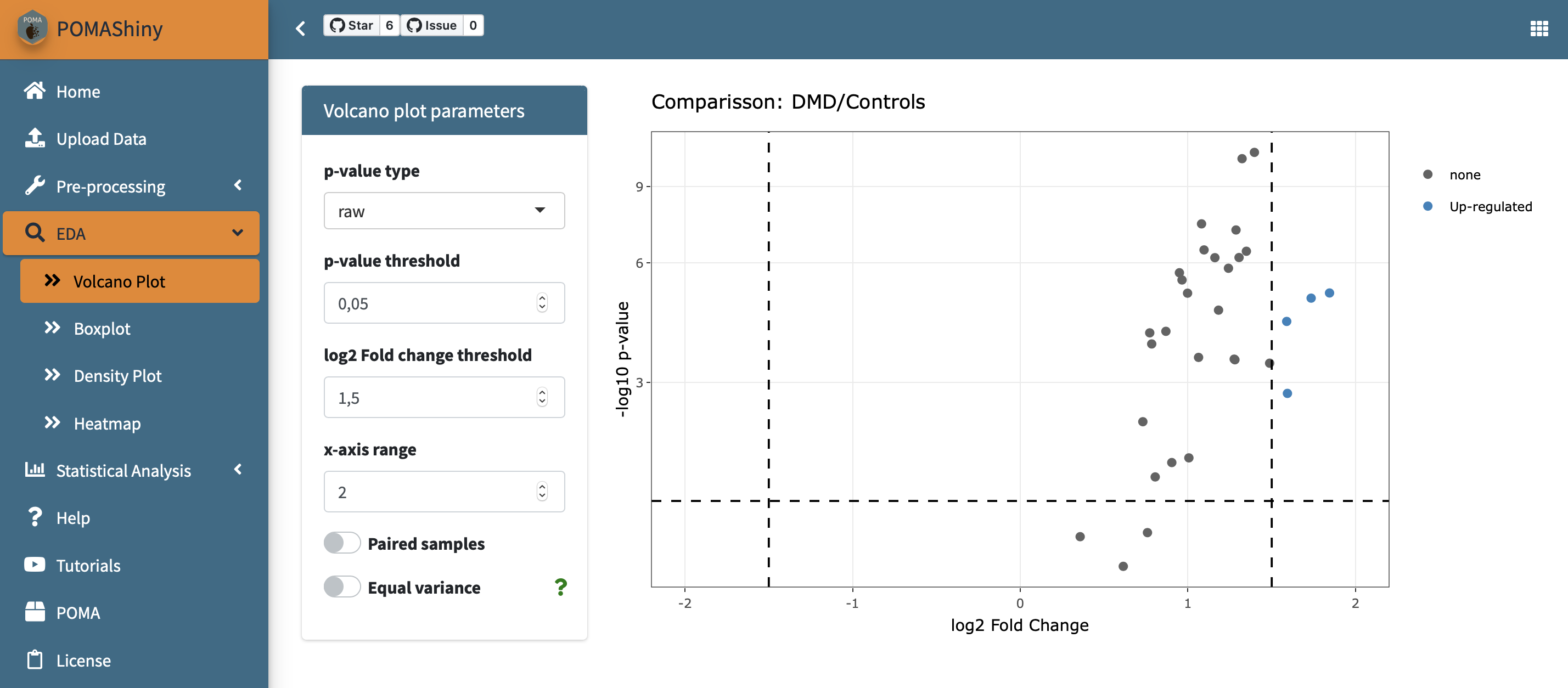

Supplement: S1 Code — In addition to the source code, the archive file contains the documentation for the installation and usage of the app and the Dockerfile to create a Docker image of POMAShiny. (ZIP) [file pcbi.1009148.s001.zip › POMAShiny-1.2.0/app/mds/pix/volcano.png]

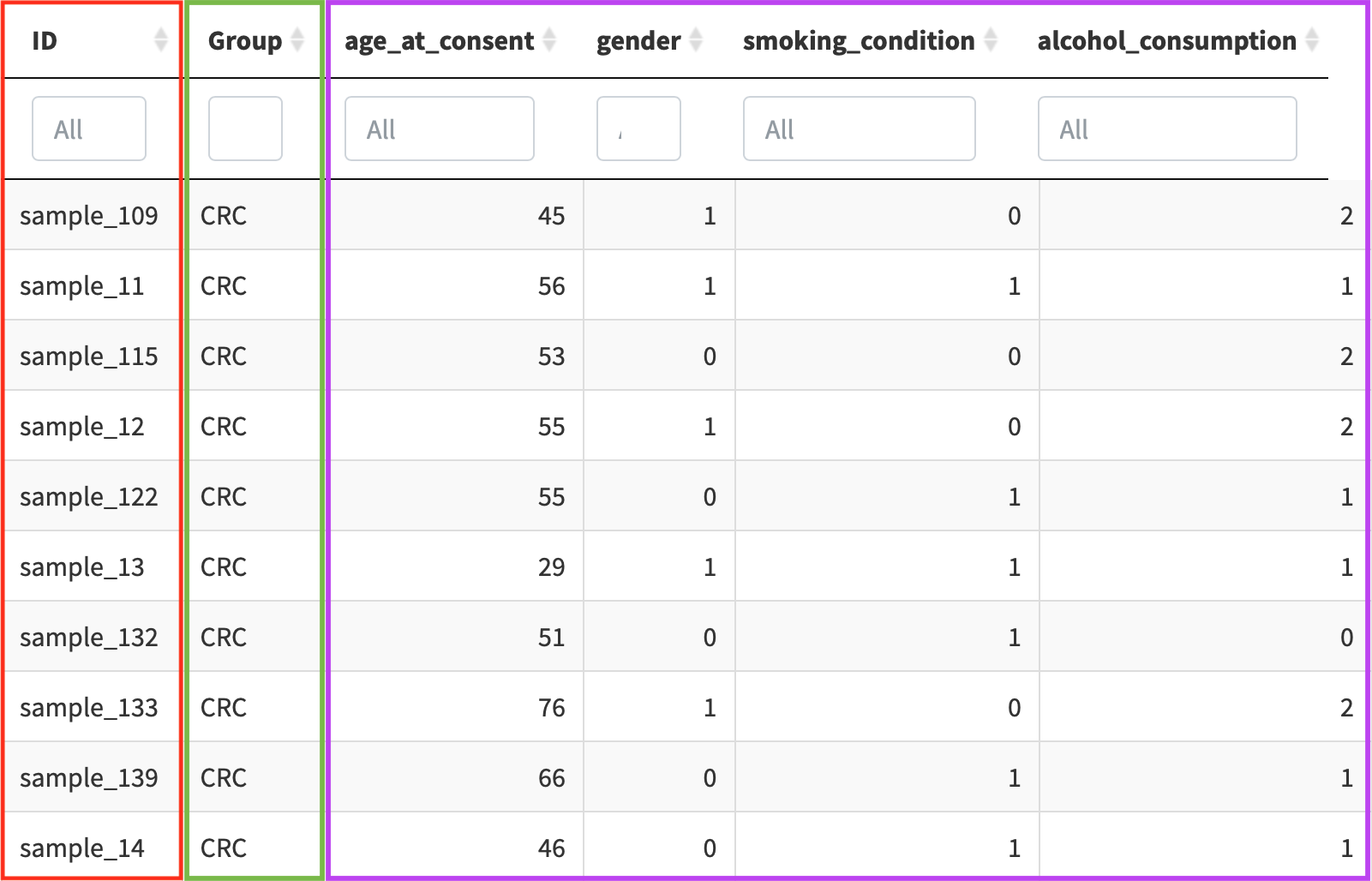

Supplement: S1 Code — In addition to the source code, the archive file contains the documentation for the installation and usage of the app and the Dockerfile to create a Docker image of POMAShiny. (ZIP) [file pcbi.1009148.s001.zip › POMAShiny-1.2.0/app/mds/pix/target.png]

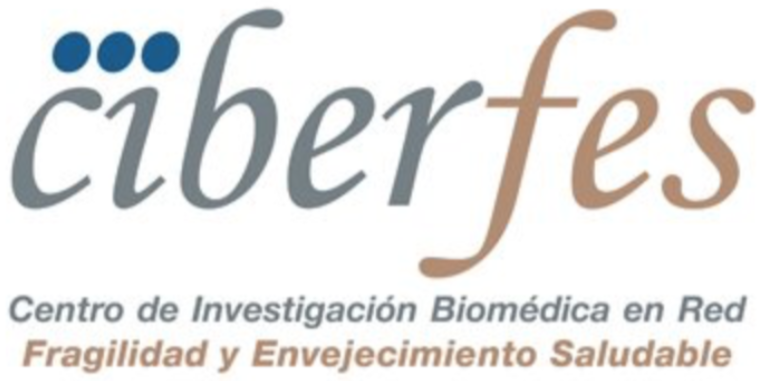

Supplement: S1 Code — In addition to the source code, the archive file contains the documentation for the installation and usage of the app and the Dockerfile to create a Docker image of POMAShiny. (ZIP) [file pcbi.1009148.s001.zip › POMAShiny-1.2.0/app/mds/pix/ciberfes.png]

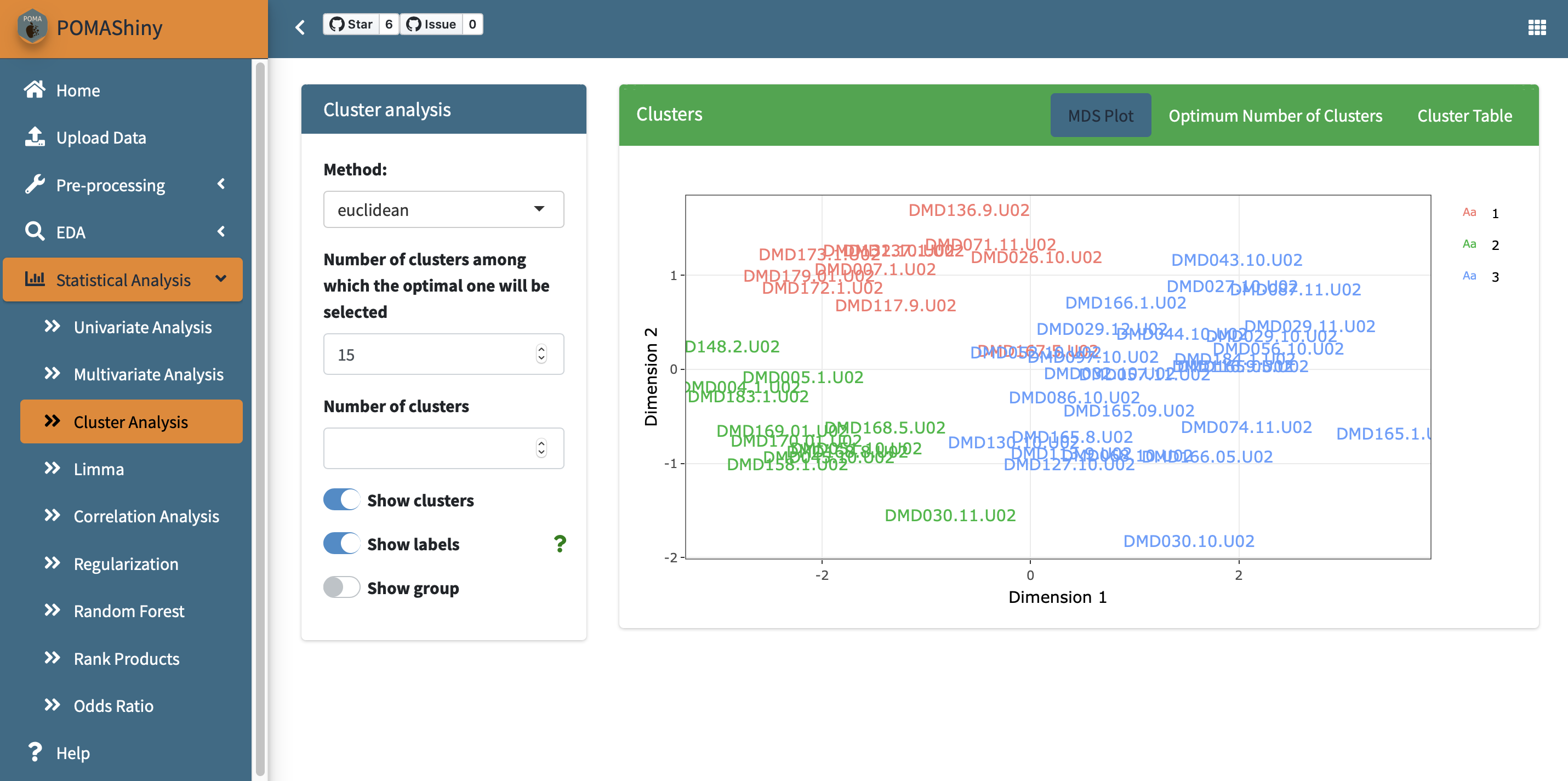

Supplement: S1 Code — In addition to the source code, the archive file contains the documentation for the installation and usage of the app and the Dockerfile to create a Docker image of POMAShiny. (ZIP) [file pcbi.1009148.s001.zip › POMAShiny-1.2.0/app/mds/pix/kmeans.png]

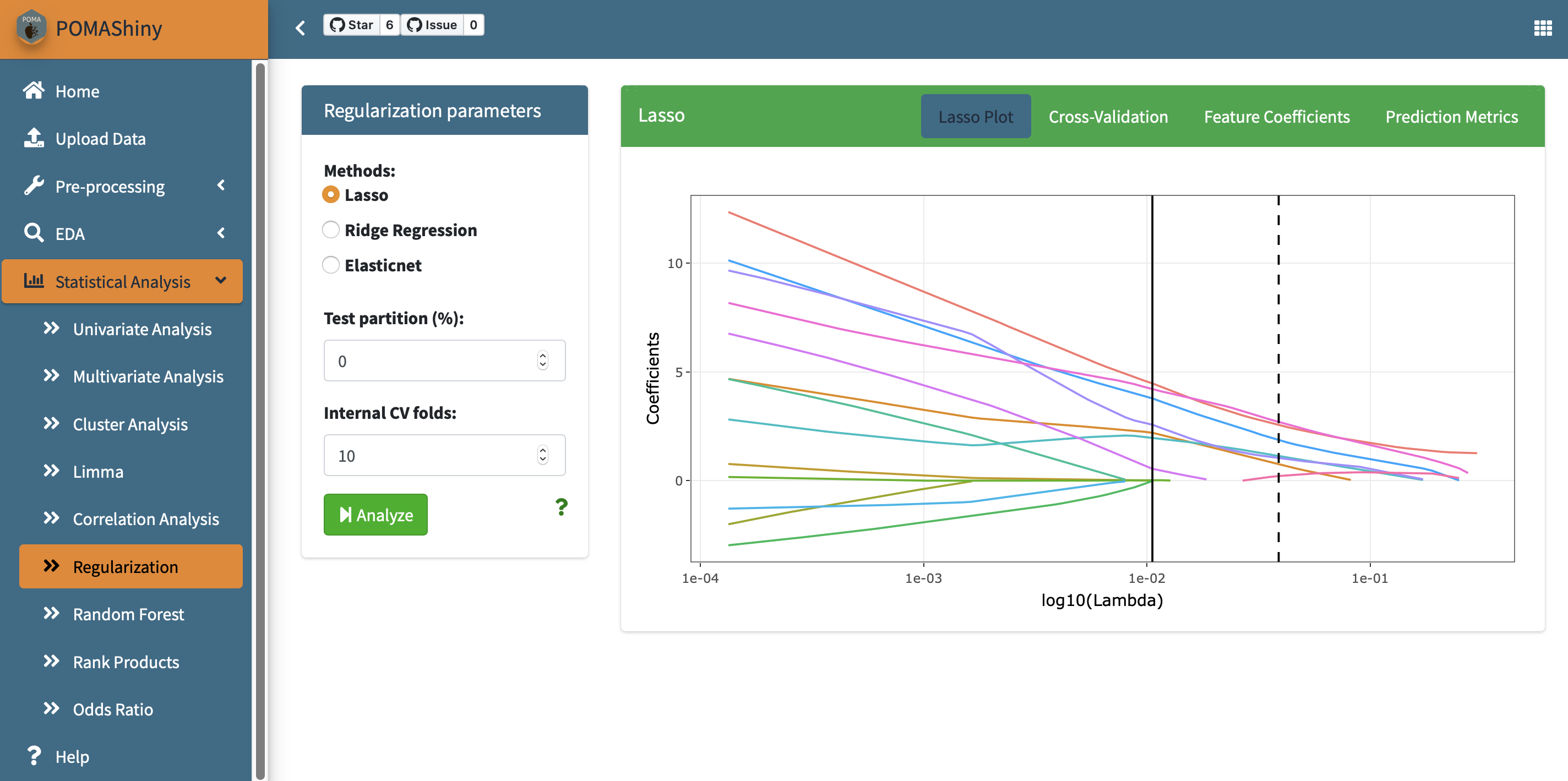

Supplement: S1 Code — In addition to the source code, the archive file contains the documentation for the installation and usage of the app and the Dockerfile to create a Docker image of POMAShiny. (ZIP) [file pcbi.1009148.s001.zip › POMAShiny-1.2.0/app/mds/pix/lasso.png]

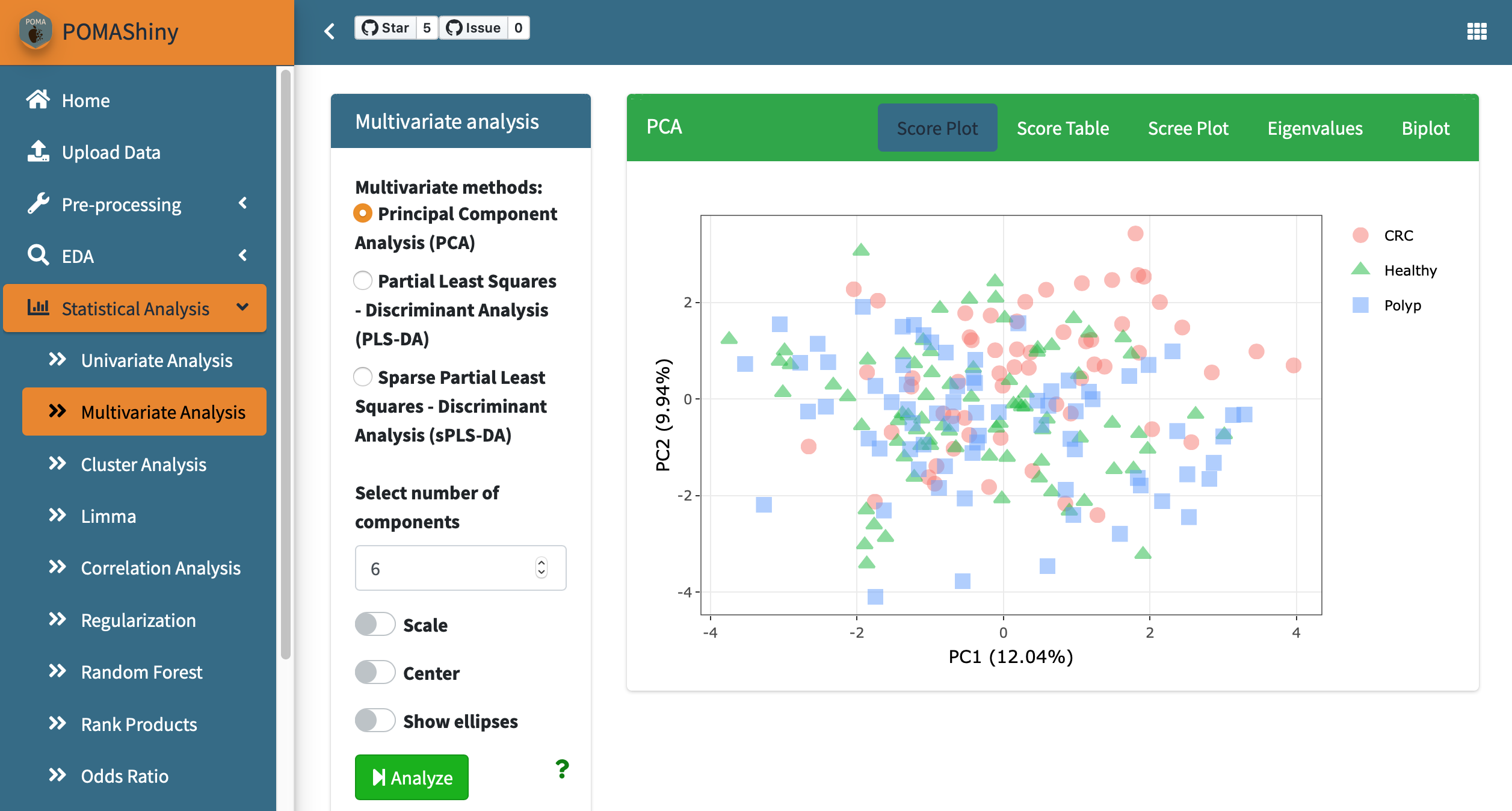

Supplement: S1 Code — In addition to the source code, the archive file contains the documentation for the installation and usage of the app and the Dockerfile to create a Docker image of POMAShiny. (ZIP) [file pcbi.1009148.s001.zip › POMAShiny-1.2.0/app/mds/pix/pca.png]

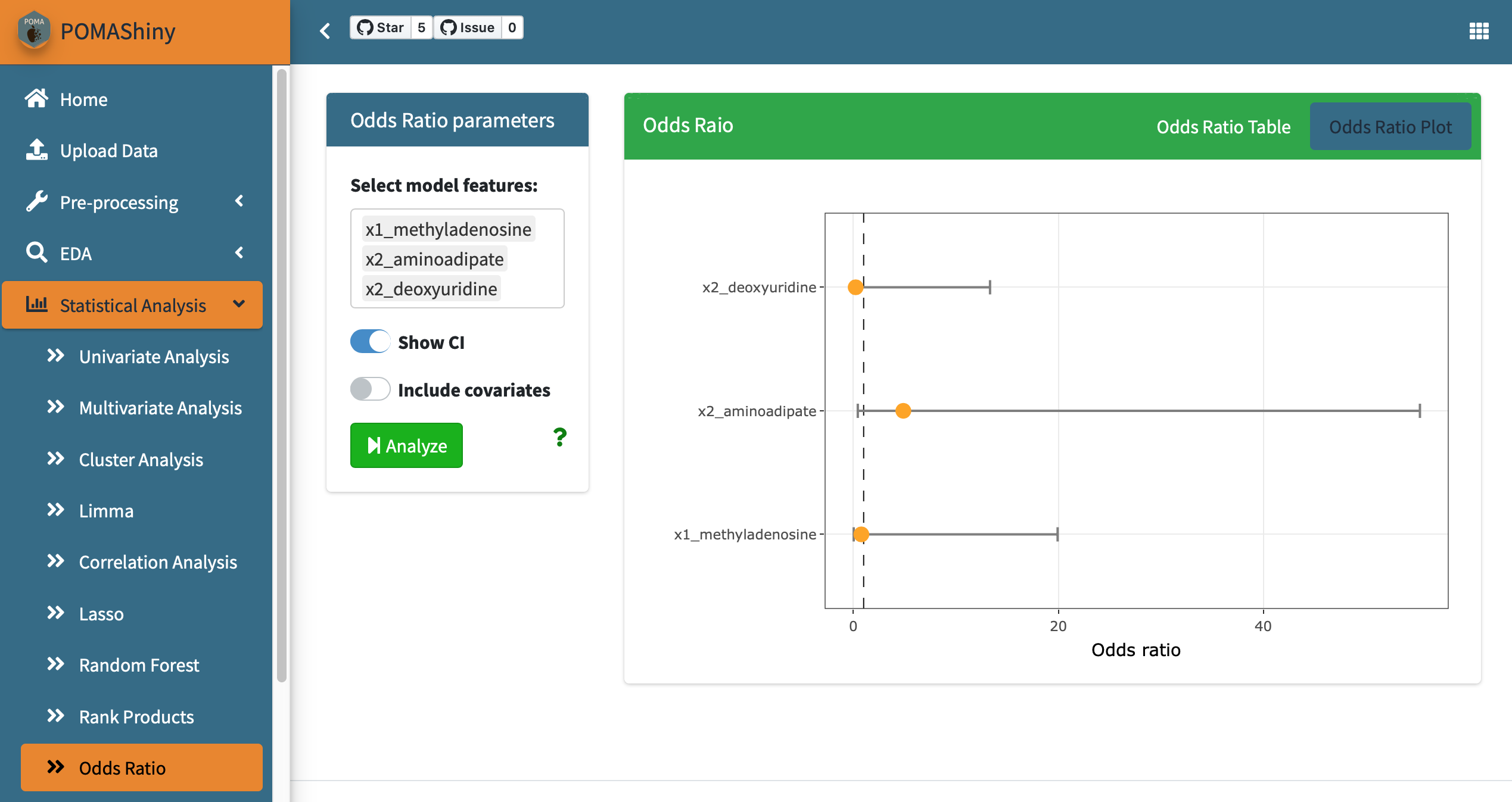

Supplement: S1 Code — In addition to the source code, the archive file contains the documentation for the installation and usage of the app and the Dockerfile to create a Docker image of POMAShiny. (ZIP) [file pcbi.1009148.s001.zip › POMAShiny-1.2.0/app/mds/pix/odds.png]

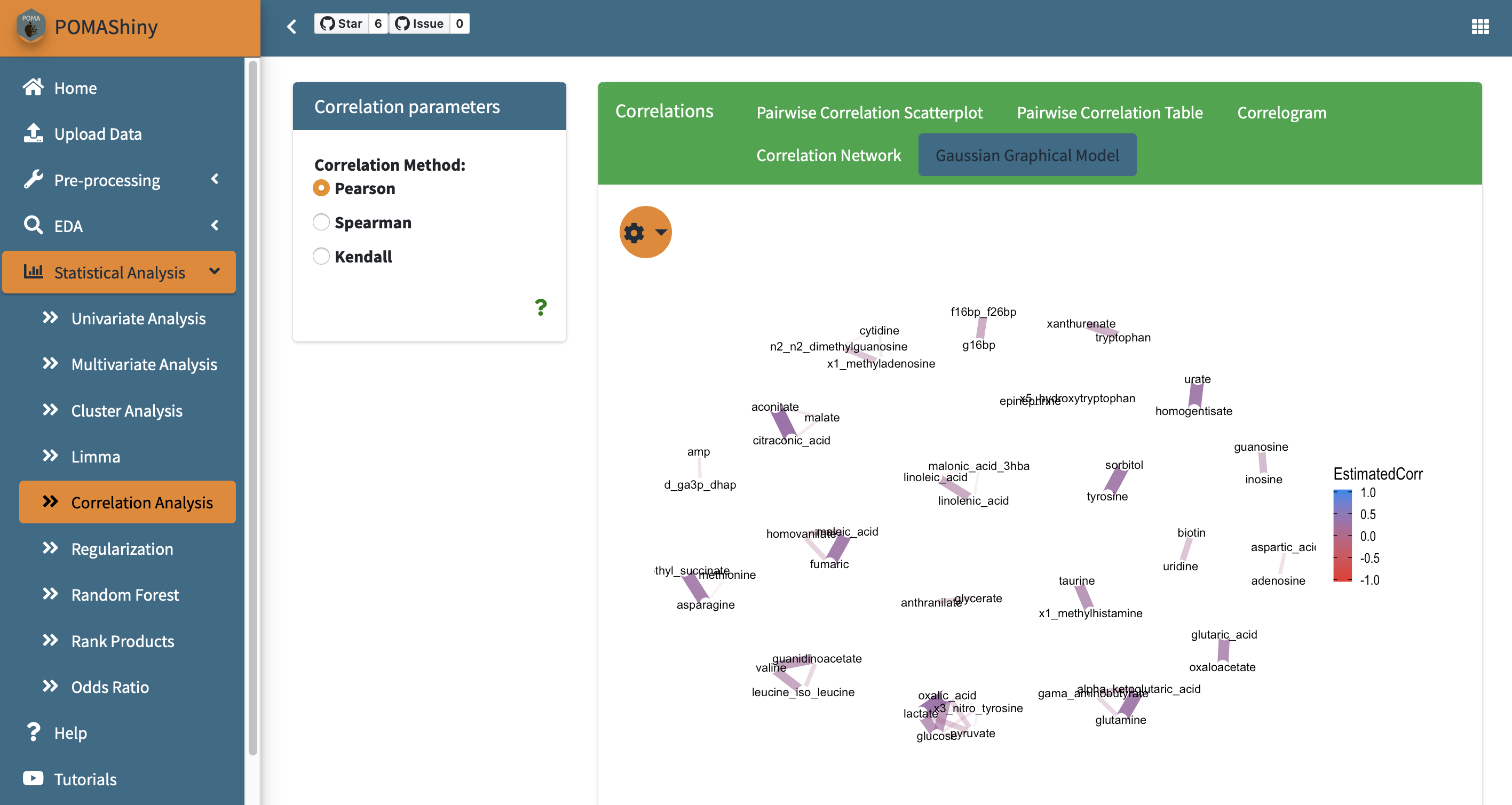

Supplement: S1 Code — In addition to the source code, the archive file contains the documentation for the installation and usage of the app and the Dockerfile to create a Docker image of POMAShiny. (ZIP) [file pcbi.1009148.s001.zip › POMAShiny-1.2.0/app/mds/pix/glasso.png]

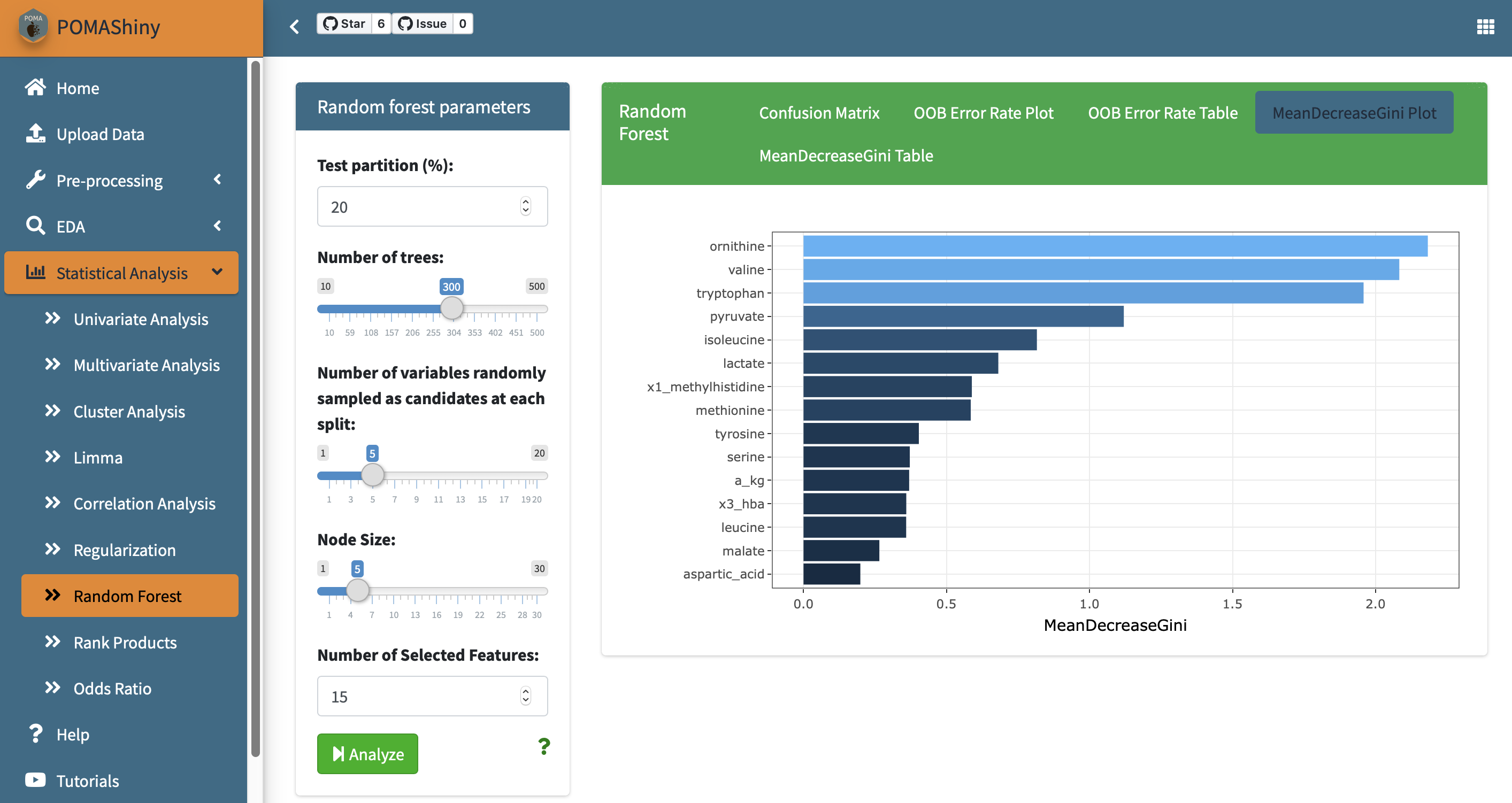

Supplement: S1 Code — In addition to the source code, the archive file contains the documentation for the installation and usage of the app and the Dockerfile to create a Docker image of POMAShiny. (ZIP) [file pcbi.1009148.s001.zip › POMAShiny-1.2.0/app/mds/pix/randomforest.png]

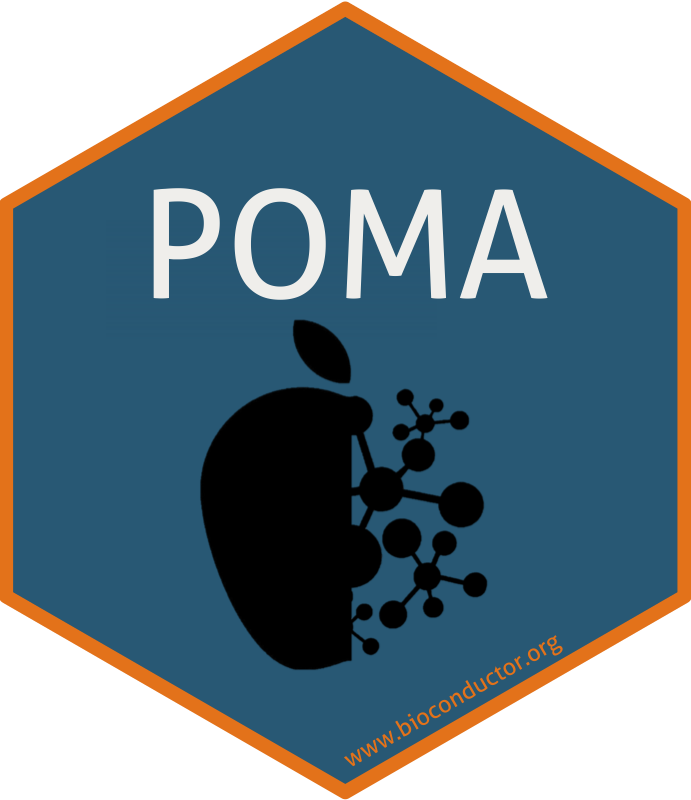

Supplement: S1 Code — In addition to the source code, the archive file contains the documentation for the installation and usage of the app and the Dockerfile to create a Docker image of POMAShiny. (ZIP) [file pcbi.1009148.s001.zip › POMAShiny-1.2.0/app/mds/pix/logo.png]

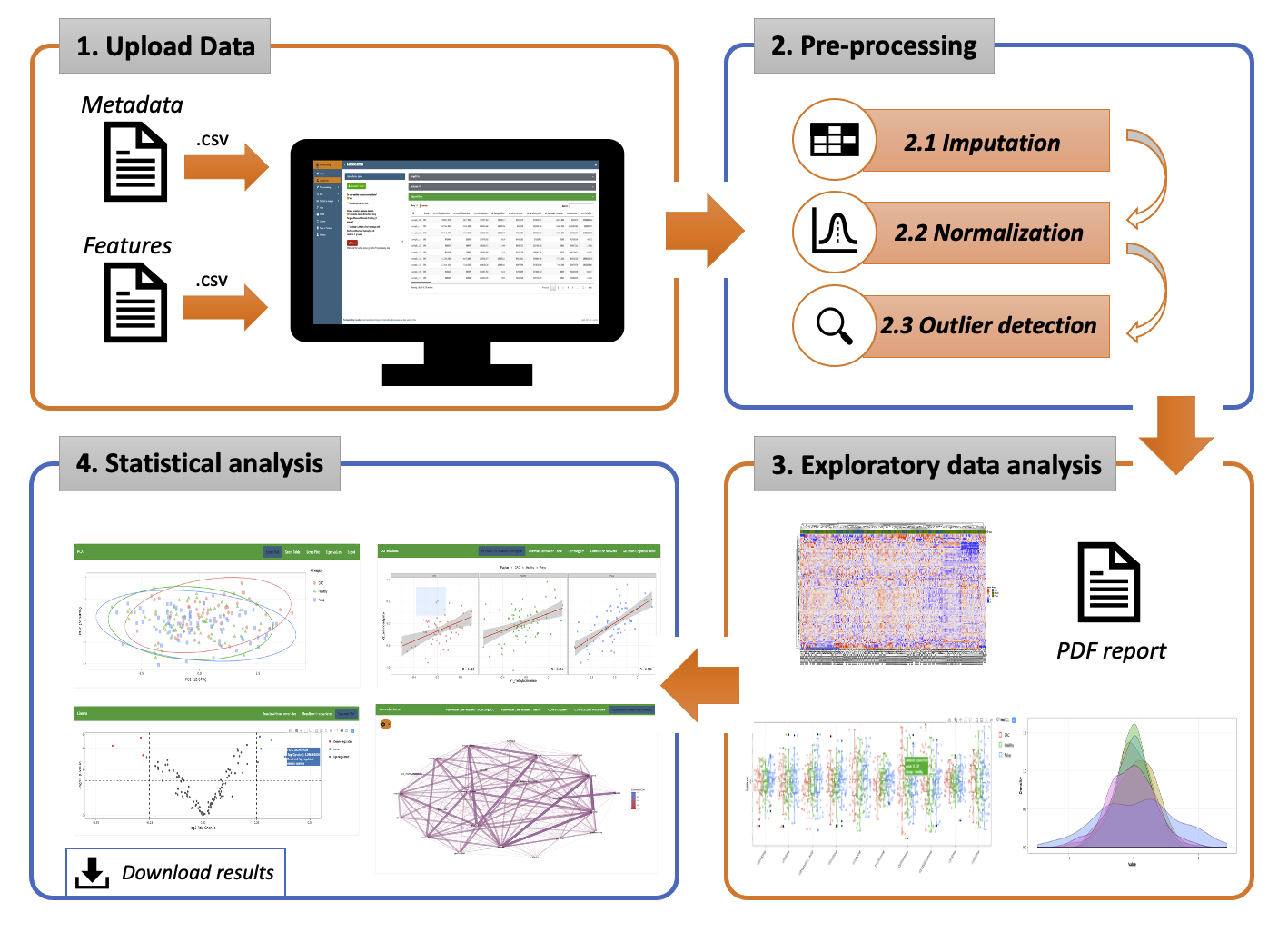

Supplement: S1 Code — In addition to the source code, the archive file contains the documentation for the installation and usage of the app and the Dockerfile to create a Docker image of POMAShiny. (ZIP) [file pcbi.1009148.s001.zip › POMAShiny-1.2.0/app/mds/pix/graphical_abstract.png]

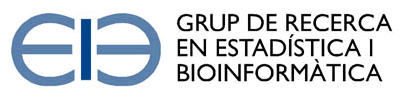

Supplement: S1 Code — In addition to the source code, the archive file contains the documentation for the installation and usage of the app and the Dockerfile to create a Docker image of POMAShiny. (ZIP) [file pcbi.1009148.s001.zip › POMAShiny-1.2.0/app/mds/pix/eib.gif]

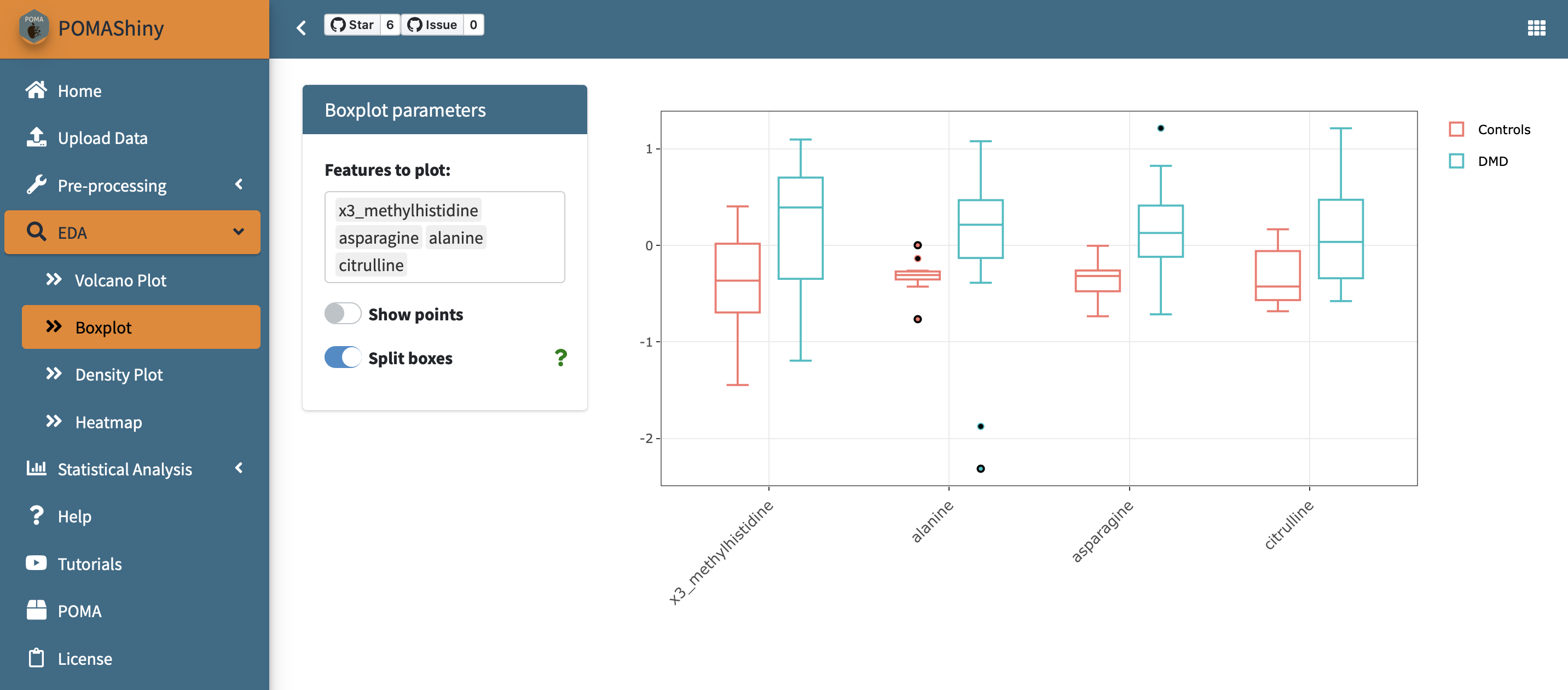

Supplement: S1 Code — In addition to the source code, the archive file contains the documentation for the installation and usage of the app and the Dockerfile to create a Docker image of POMAShiny. (ZIP) [file pcbi.1009148.s001.zip › POMAShiny-1.2.0/app/mds/pix/boxplot.png]

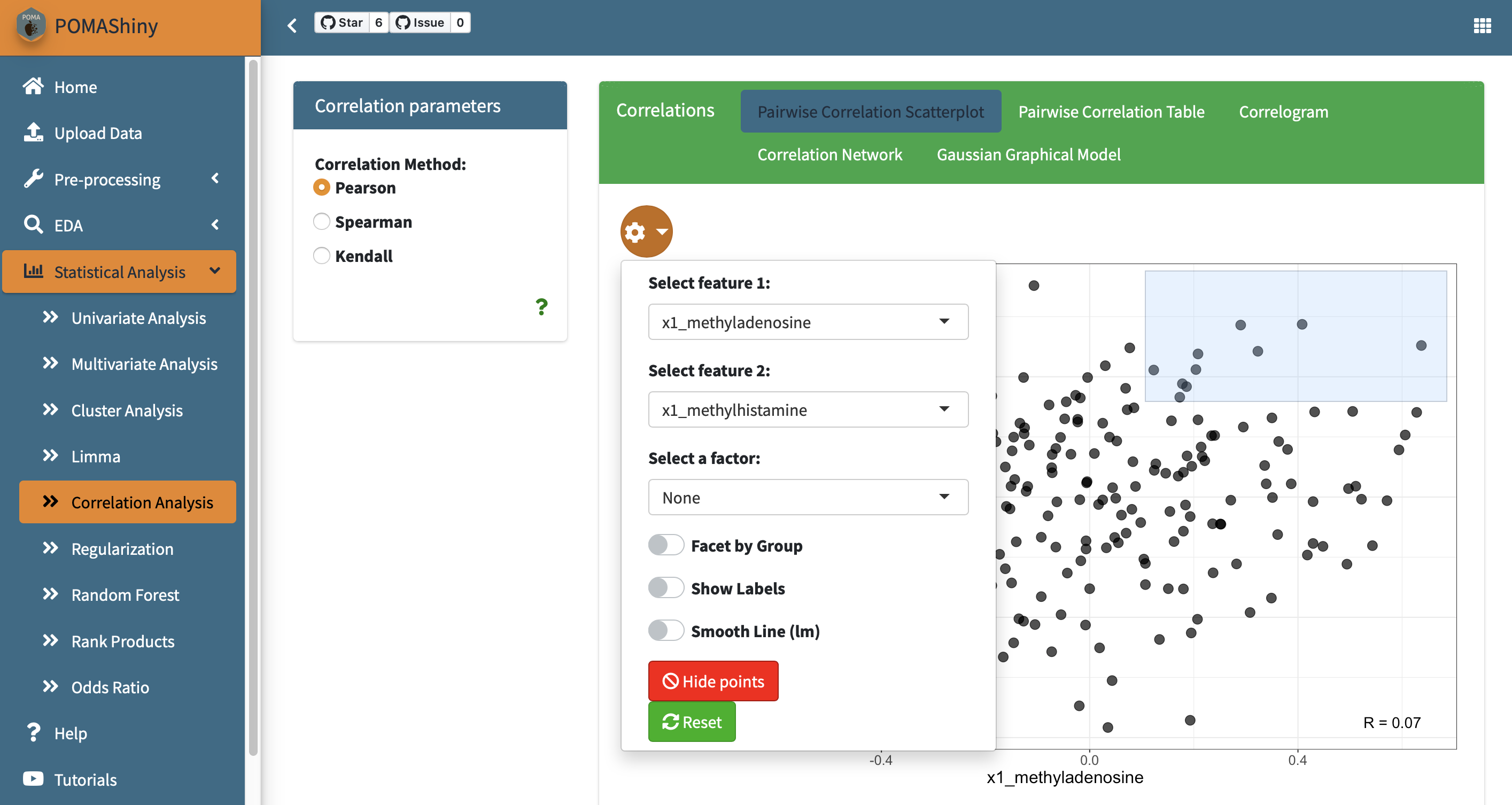

Supplement: S1 Code — In addition to the source code, the archive file contains the documentation for the installation and usage of the app and the Dockerfile to create a Docker image of POMAShiny. (ZIP) [file pcbi.1009148.s001.zip › POMAShiny-1.2.0/app/mds/pix/scatter.png]

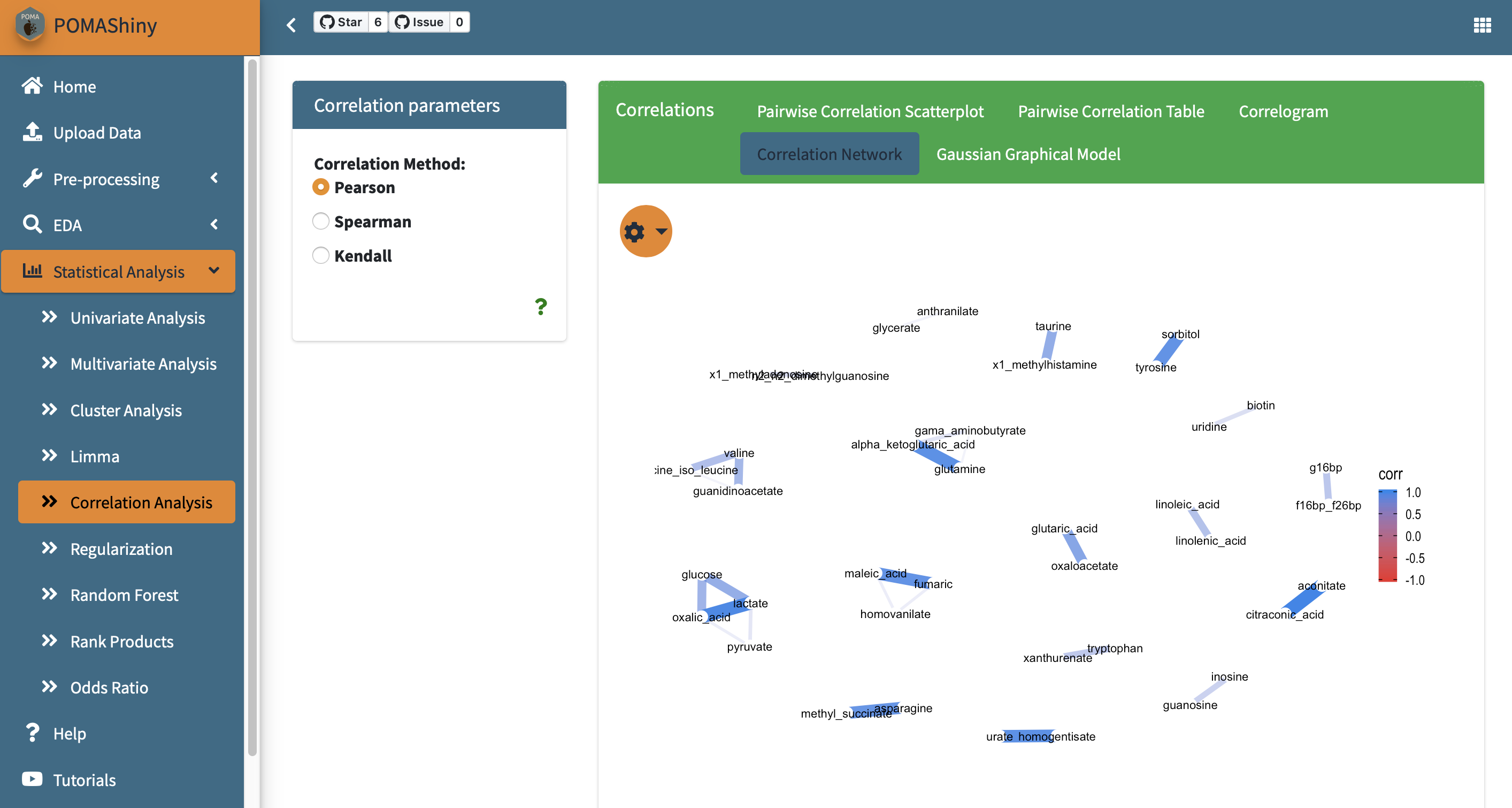

Supplement: S1 Code — In addition to the source code, the archive file contains the documentation for the installation and usage of the app and the Dockerfile to create a Docker image of POMAShiny. (ZIP) [file pcbi.1009148.s001.zip › POMAShiny-1.2.0/app/mds/pix/network.png]

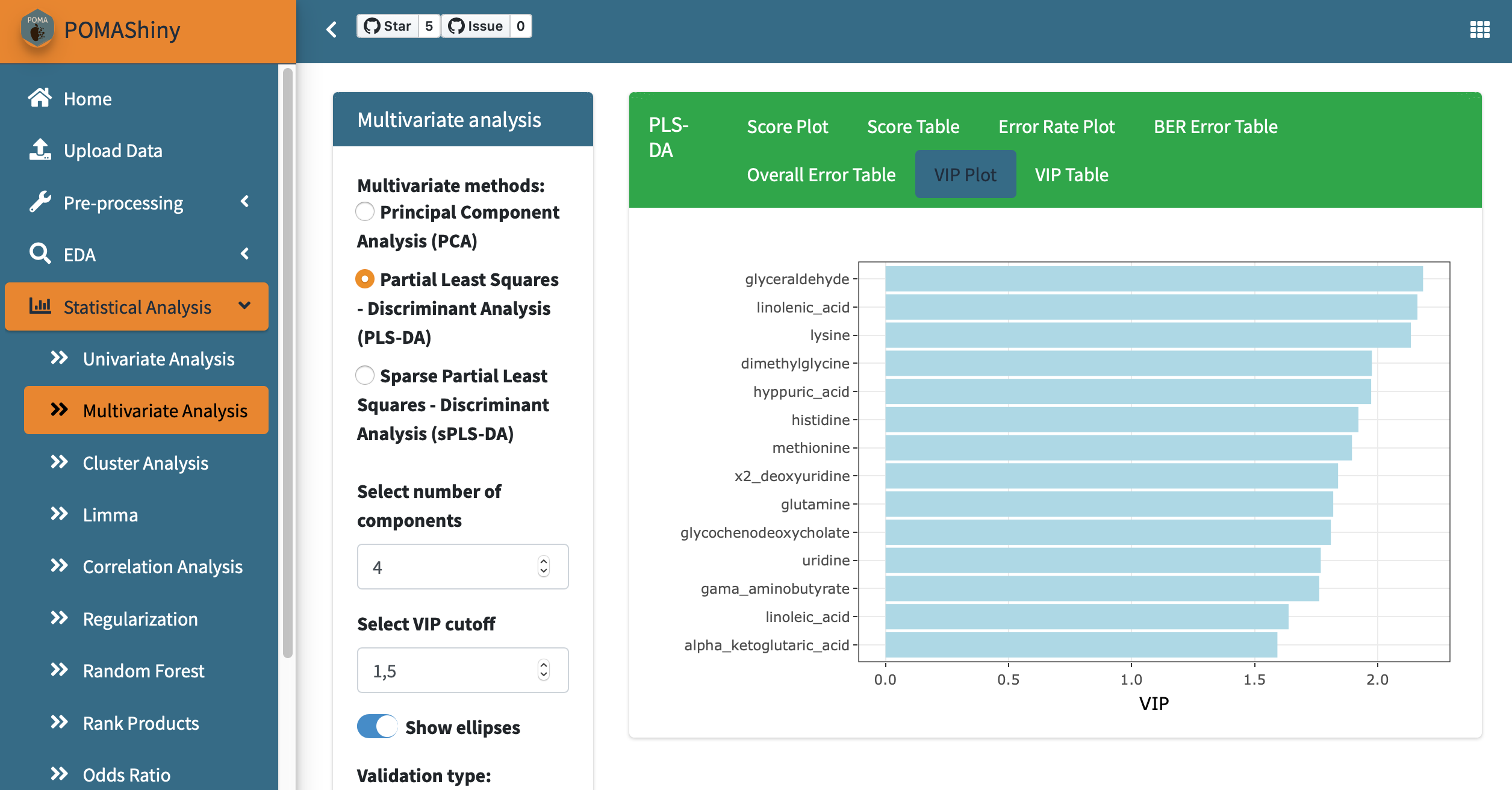

Supplement: S1 Code — In addition to the source code, the archive file contains the documentation for the installation and usage of the app and the Dockerfile to create a Docker image of POMAShiny. (ZIP) [file pcbi.1009148.s001.zip › POMAShiny-1.2.0/app/mds/pix/plsda.png]

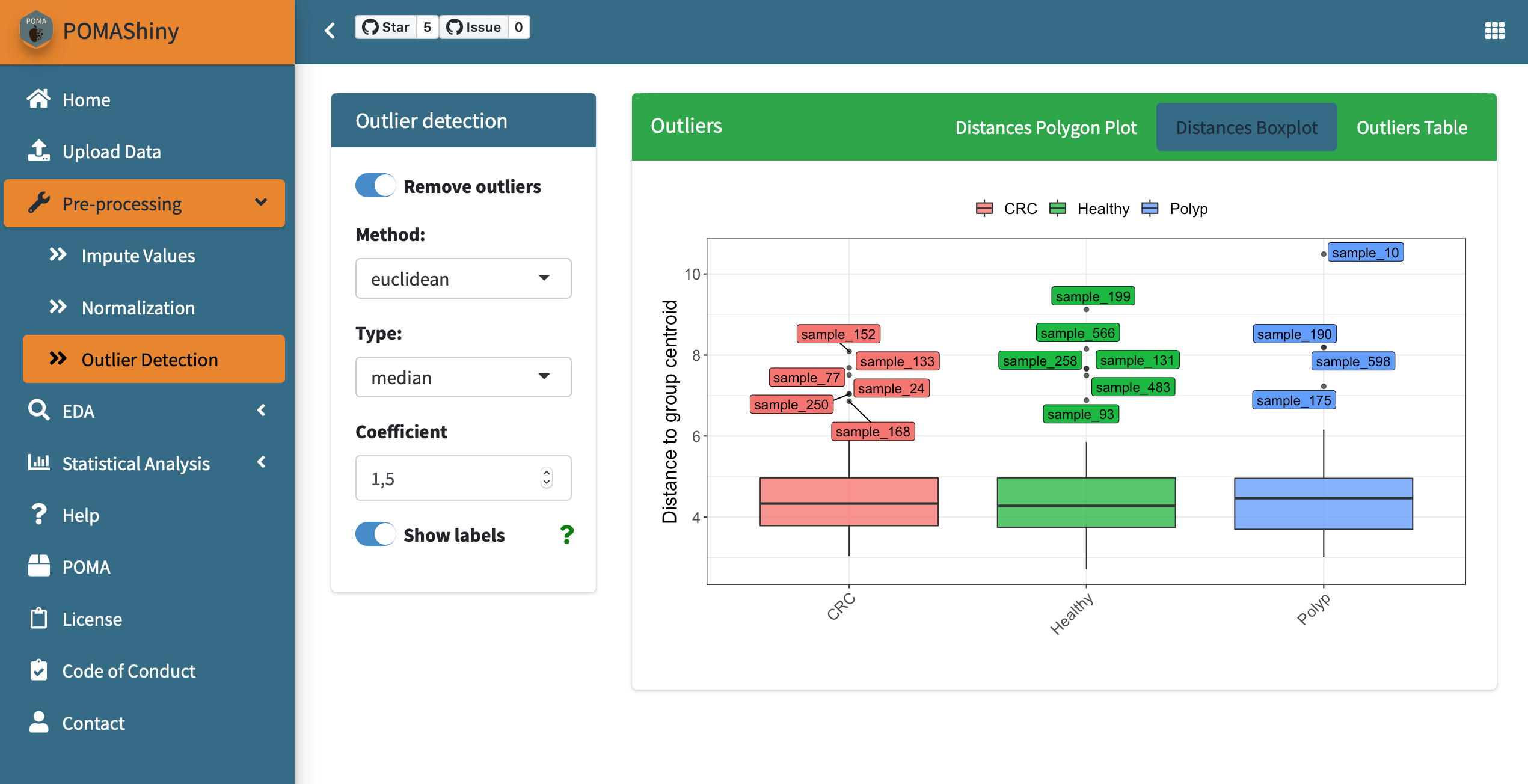

Supplement: S1 Code — In addition to the source code, the archive file contains the documentation for the installation and usage of the app and the Dockerfile to create a Docker image of POMAShiny. (ZIP) [file pcbi.1009148.s001.zip › POMAShiny-1.2.0/app/mds/pix/outliers.png]

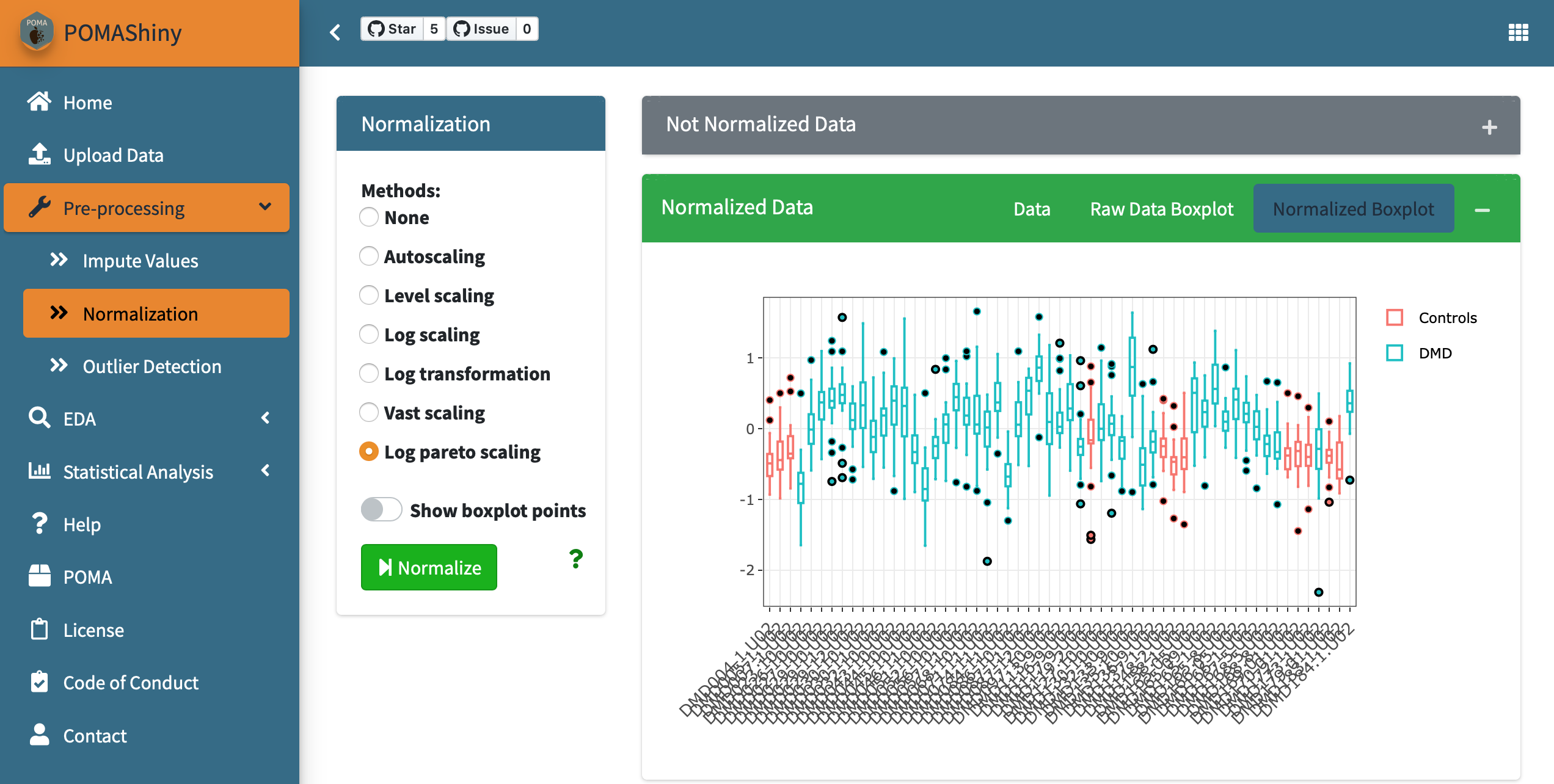

Supplement: S1 Code — In addition to the source code, the archive file contains the documentation for the installation and usage of the app and the Dockerfile to create a Docker image of POMAShiny. (ZIP) [file pcbi.1009148.s001.zip › POMAShiny-1.2.0/app/mds/pix/normalization.png]

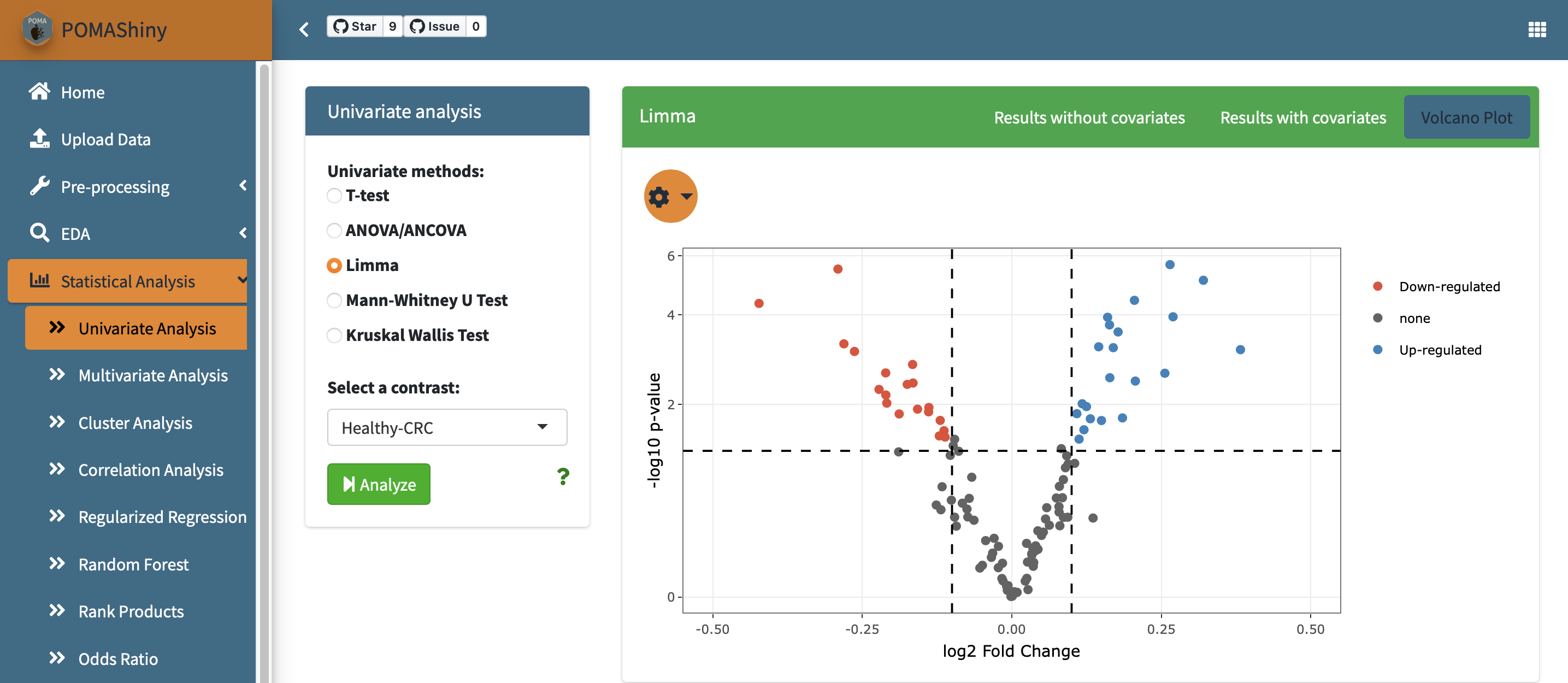

Supplement: S1 Code — In addition to the source code, the archive file contains the documentation for the installation and usage of the app and the Dockerfile to create a Docker image of POMAShiny. (ZIP) [file pcbi.1009148.s001.zip › POMAShiny-1.2.0/app/mds/pix/limma.png]

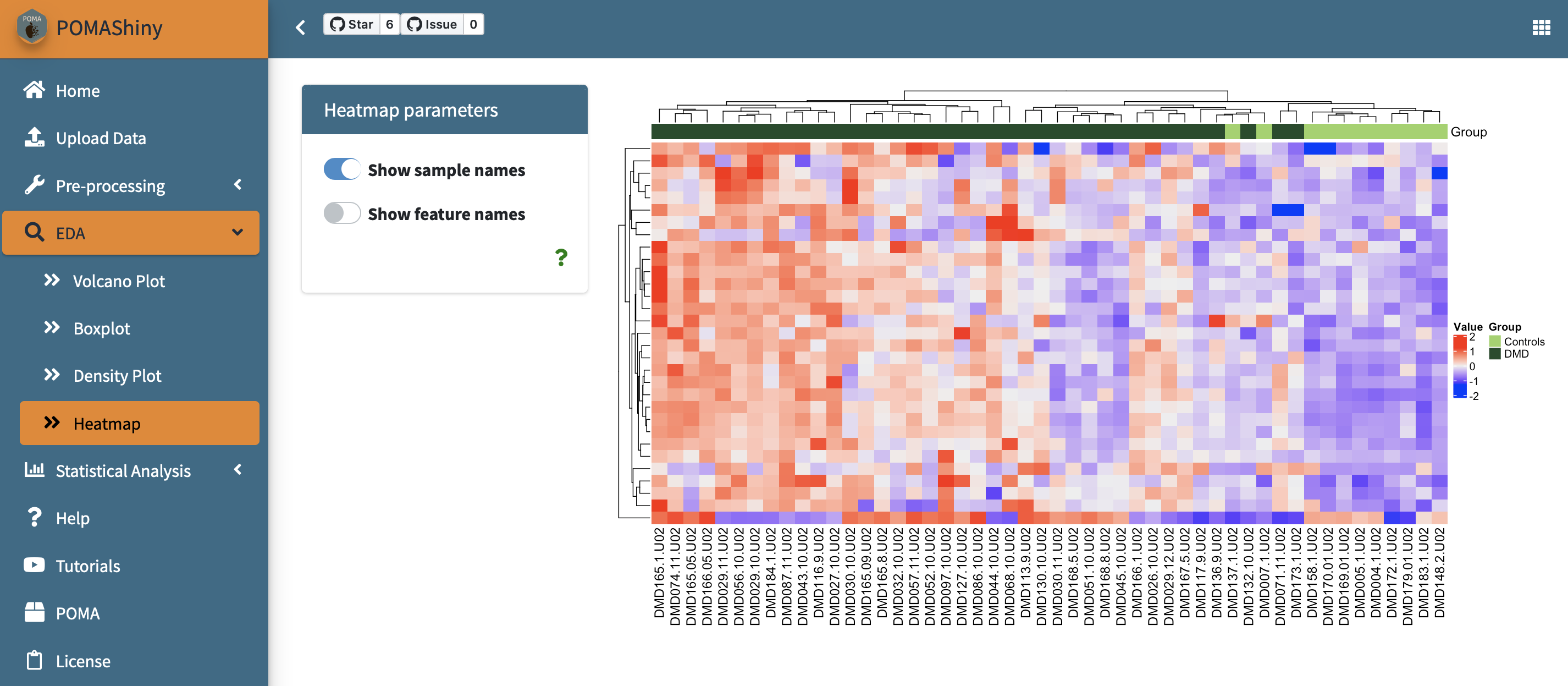

Supplement: S1 Code — In addition to the source code, the archive file contains the documentation for the installation and usage of the app and the Dockerfile to create a Docker image of POMAShiny. (ZIP) [file pcbi.1009148.s001.zip › POMAShiny-1.2.0/app/mds/pix/heatmap.png]

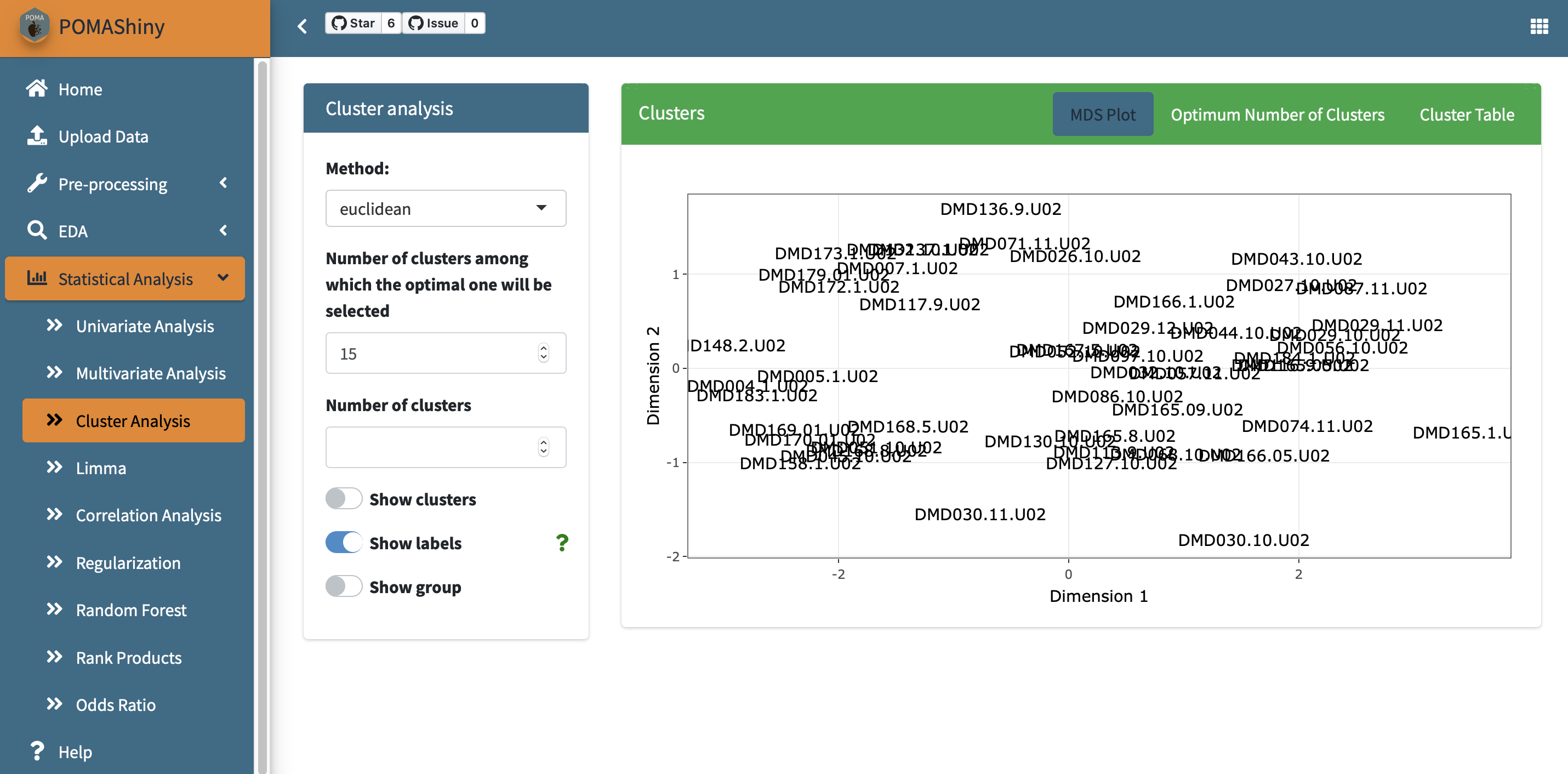

Supplement: S1 Code — In addition to the source code, the archive file contains the documentation for the installation and usage of the app and the Dockerfile to create a Docker image of POMAShiny. (ZIP) [file pcbi.1009148.s001.zip › POMAShiny-1.2.0/app/mds/pix/mds.png]
