# Supplementary material for: POMAShiny: A user-friendly web-based workflow for metabolomics and proteomics data analysis
Source: PLoS Comput Biol. 2021 Jul 1;17(7):e1009148. doi: 10.1371/journal.pcbi.1009148 (PMC8279420; doi:10.1371/journal.pcbi.1009148)
Supplement: Attachment — Submitted filename: POMAShiny_response_to_reviewers.pdf [file pcbi.1009148.s003.pdf]

Point-by-point Response to Reviewers

Editors:

We are grateful to the editors for considering our work for publication. We have modified the manuscript in accordance with the reviewers' comments and suggestions in order to clarify some aspects and improve its quality so that it is suitable for the PLOS Computational Biology journal. We believe this new version reflects our work better and includes some new content and features that improve both the readability of the manuscript and the usability of the application.

Furthermore, we would like to request that Prof. Dr Francesc Carmona-Pontaque (*fcarmona@ub.edu*) be added to the author list for his valuable contributions in the review process and for his contributions to the conceptualization of this work, especially in the development of the outliers detection method provided in the application, which had not been duly considered at the first submission.

Reviewer 1:

We thank the reviewer for his/her thoughtful review of our work, constructive comments and kind words. We have reviewed the manuscript thoroughly and addressed any errors or suggestions we came across with the help of the reviewers.

<u>Major issues</u>

1) The authors state that their software is designed for MS data analysis but the data input format are simple tabulator delimited text files (same as one would get from Microarray experiments or other quantitative large scale assays). The tool does not support raw MS data handling or import nor does it provide any methodology which is specific to MS data (such as combining feature representing peptides of the same protein in proteomics or grouping of features representing ions of the same compound for metabolomics). The authors should either put less emphasis on MS data analysis and describe their tool as a user interface for data analysis methods commonly used across all areas and platforms used in computational biology, or should provide indeed MS specific functionality.

We agree that POMAShiny provides different generic features and methods that can be used in other quantitative large-scale assays and thank the reviewer for pointing this out. The POMAShiny application was called an "MS data analysis tool" because the internal data structures used in POMA (and consequently in POMAShiny) are designed specifically for MS data.

After considering the reviewer’s comments, we realized that the methodologies provided in POMAShiny focus more on statistical analysis of metabolomics and proteomics (regardless of the technique used to analyse them) than on the MS data analysis (especially in the preprocessing step (ref. 19–26)).

Therefore, we have changed “MS” to “metabolomics and proteomics” in the title and throughout the manuscript, putting less emphasis on MS data, as the reviewer suggests. We believe that this change improves the comprehension of the manuscript and makes the tool more extensible and in line with the applications and methods it provides.

Additionally, although POMAShiny is focused on statistical analysis, we have added a new feature to provide users with at least a minimum level of MS functionality. We have added a new option in the “data upload panel” that allows users to combine different features that are part of the same “entity”. This functionality has been well documented in both the general “Help” panel and the individual help panel. This optional feature is powered by the R/Bioconductor MSnbase package and it allows features to be combined in cases such as when the data contain different peptides of the same protein or different ions representing the same compound.

If users enable this option, they will be prompted to upload an additional file (CSV) called “grouping file”, indicating which features should be combined. This new functionality offers five different methods for performing this task and allows users to assess the quality of the combination by providing a downloadable table with the coefficients of variation of the combined features.

An additional example dataset, available in the GitHub repository, has also been included in order to provide users with a template for each file required in POMAShiny (target file, features file and grouping file).

This new feature has also been detailed in the manuscript between lines 94 and 100. This section now reads:

“Furthermore, POMAShiny provides a function that allows users to combine different features that are part of the same entity. This optional operation can be very useful when the data contain different peptides that are part of the same protein or different ions representing the same compound. If users enable this option, a “grouping file” (CSV) indicating which features should be combined will be required. Several methods for performing this task are provided in POMAShiny, as well as the ability to download a table with the coefficients of variation of those combined features.”

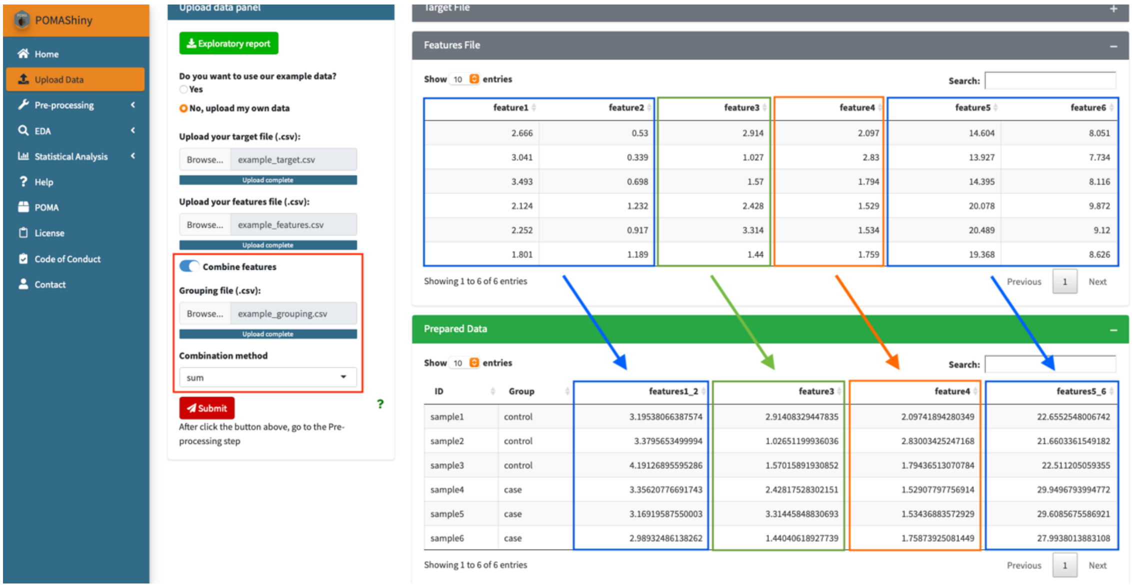

Figure 1. The structure of the feature combination option in POMAShiny. In this picture it is shown how this functionality works by combining features 1 and 2, and features 5 and 6, in the example data set. The example data set provided in the POMAShiny GitHub repository has been used to create this figure.

2) Also, the authors should consider to emphasize more the integration of methodology provided by the POMA package which reduces the need for transformation/conversion between data types and objects.

We thank the reviewer for pointing this out. We have clarified this concept between lines 43 and 47. Now it reads:

"[...] integration and interoperability with other workflows, such as the RforMassSpectrometry initiative (RforMassSpectrometry.org), which provides the data structures used by the POMA package. Therefore, users can perform the spectral data processing and other routine MS workflow operations using the RforMassSpectrometry complementary packages, and then easily migrate to POMA to perform the statistical analysis without changing the data structure."

3) The authors need to compare their tool to other web-based tools such as iSEE or W4M.

We agree that a comparative section is mandatory and thank the reviewer for pointing this out. As the reviewer suggests, a section called "*Comparison with existing tools*" has been added to the manuscript (lines 363–406). This new section is focused on a comparison of the POMAShiny, W4M and MetaboAnalyst tools.

In addition to this new section, a detailed table comparing the aforementioned tools has been added to the manuscript for a faster, more understandable and more visual comparison (see Table 2).

The main points explained in the comparison section are:

- The interactive visualization and exploratory data analysis
- Preprocessing options (especially the outlier treatment)
- The ability to include covariates in the analysis and the statistical methods provided
- The ability to create random test and train sets in the offered predictive modelling strategies

4) The written english of the document needs to be improved (see also minor issues below).

We agree that an accurate review of the written English was needed and thank the reviewer for pointing this out. The written English has been thoroughly reviewed and improved throughout the manuscript.

5) The authors state that POMAShiny allows more advanced users to replicate an analysis in R (without the web frontend). This would require that the actual commands used to perform the analysis are provided/shown to the user. Is this the case? If not this argument should be removed.

We thank the reviewer for pointing out this confusion. With this argument we meant that since POMAShiny uses the POMA functions, POMA users can reproduce exactly the same outputs as those obtained with POMAShiny.

However, thanks to the reviewer's comment, we have come to realize that the term "replicate" can refer to providing users with the exact commands that are running at each step in POMAShiny. Unfortunately, this option is not available in POMAShiny, partly because it is a complex issue that has not yet been fully resolved in Shiny applications. Therefore, we apologize for our mistake and agree with the reviewer that it is best to remove this sentence (line 39) so as not to confuse users.

Alternatively, if users want to "reproduce" or extend the analysis in R (without the Shiny interface), they have at their disposal three different well-documented vignettes on the POMA package website in order to follow the step-by-step analysis (https://pcastellanoescuder.github.io/POMA/). In addition, in the "Help" panel of the POMAShiny application, users can find each homologous POMA function for each step of the analysis conducted with POMAShiny. However, we know that this is not the optimum scenario, as this might not replicate exactly

the same analysis, and for this reason we have decided to remove the sentence highlighted by the reviewer.

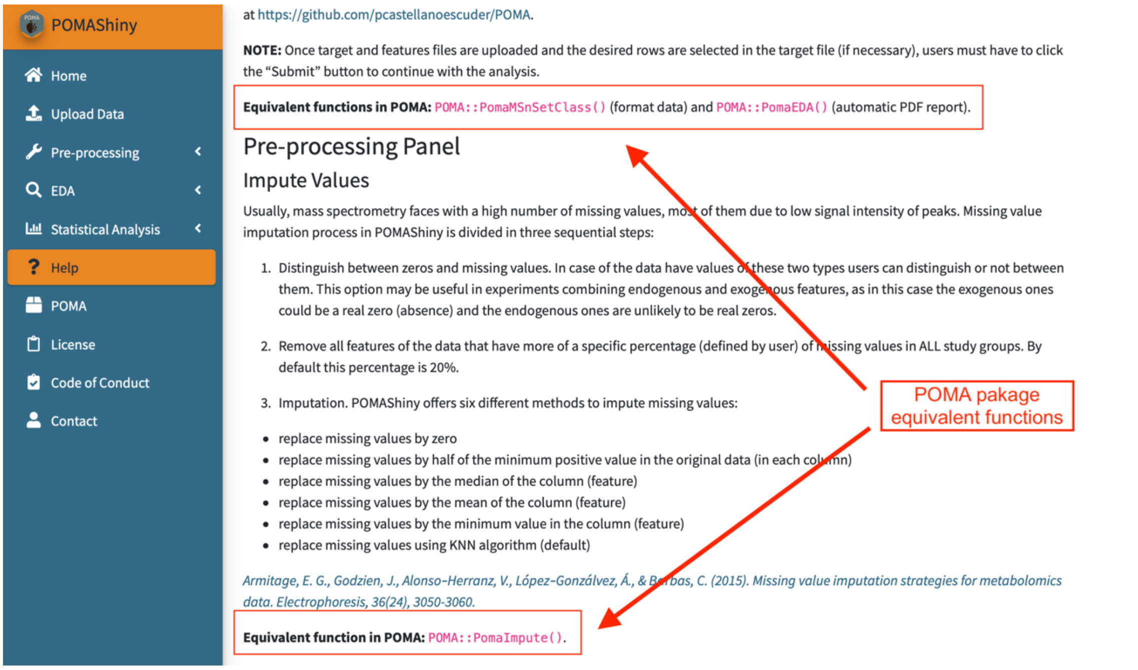

Figure 2. POMA package equivalent functions provided in the POMAShiny "Help" panel.

6) The authors should consider to provide the available algorithms/methods for the statistical methods as a table and to emphasize more on what is available in their software and less on the description on the various methods and why they should be used. The main scope of the manuscript should be the presentation of their software and less the description of data analysis algorithms.

We agree that a table showing all the statistical methods provided is needed and thank the reviewer for pointing this out. We have added a table with a classification of the statistical methods provided by the POMA and POMAShiny tools (Table 1, page 7). In addition, in line with the reviewer's suggestion, we have tried to clarify in each section of the manuscript what is provided in POMAShiny instead of the methodology itself (lines 211–215, 282–284 and 309–310).

<u>Minor issues</u>

7) The provided docker pull command does not work. Only "docker pull pcastellanoescuder/pomashiny:release1.0.0" works. Authors should update/fix that.

We thank the reviewer for pointing this out. The Docker container has been updated, including all changes made in this revision. The Docker pull instruction has also been changed to: "*docker pull pcastellanoescuder/pomashiny:v.1.1.14*".

8) lines 35-38: It is not clear how POMA integrates with packages from RforMassSpectrometry as they mostly represent raw MS data (i.e. spectra data) and POMA works exclusively on "feature tables" (i.e. quantified MS data). The authors should clarify this.

We thank the reviewer for pointing this out. We have clarified this concept between lines 43 and 47. Now it reads:

"[...] integration and interoperability with other workflows, such as the RforMassSpectrometry initiative (RforMassSpectrometry.org), which provides the data structures used by the POMA package. Therefore, users can perform the spectral data processing and other routine MS workflow operations using the RforMassSpectrometry complementary packages, and then easily migrate to POMA to perform the statistical analysis without changing the data structure."

9) lines 44-46: it is not clear to the reviewer how the use of POMA and other packages facilitates contribution of other users/developers. If there are different contributors to the package/source code, the authors should provide this information, otherwise I would suggest to remove/reformulate this sentence.

There are no other contributors in POMA/POMAShiny. This statement meant that the "readability" of the code as well as the good practices used (git, tests, etc.) facilitated the contribution of users. However, thanks to the reviewer's comment, we realized that this statement could lead to confusion, so we have removed it.

10) line 81: what is meant by "simplifying the indexation procedures among data". The authors need to reformulate.

We thank the reviewer for pointing this out. This statement referred to the advantages of OOP programming, which reduces the errors arising from table conversion or type/object adaptations in order to use one method or another. Since this referred to the back-end structure of POMA and not so much to the main POMAShiny tool, we decided to remove this statement so as not to confuse users.

11) Outlier detection: the authors should emphasize less on why outlier detection is important and describe more/better how this can be achieved with their tool.

We agree that some clarification is needed here and thank the reviewer for pointing this out. In accordance with the reviewer's comment, we have added a detailed explanation of the outlier detection methodology provided in POMAShiny and how to use it, focusing more on our software. This concept has been added between lines 155 and 161. This section now reads:

"Here, we propose an ad hoc multivariate outlier detection method based on the Euclidean distances among observations and their distances to each group centroid in a two-dimensional space (maximum, manhattan, canberra and minkowski distances are also available). Once the distances are computed, the classical univariate outlier detection formula $Q3 + x * IQR$ *is used to detect multivariate group-dependent outliers using the computed distances to each group centroid (x; the higher this value, the less sensitive the method is to outliers)."*

12) lines 330-331: The authors claim that their tool allows "...making data analysis process more accessible to a wide range of researchers not so familiar with programming and/or statistical fields." This might well be correct, but especially for the target users (i.e. users less experienced with the provided statistical methods) a more comprehensive tutorial and links to descriptions of that methods is needed. Otherwise users can easily get mislead by wrong results if inappropriate methods are used (or if they are used incorrectly).

We thank the reviewer for pointing this out. Currently, both the general "Help" and individual help tabs in each panel provide detailed explanations with images and screenshots on how to carry out the different analyses. In addition, different links to publications and materials with extensive information on the POMAShiny methods' are provided. Finally, some more examples and detailed information on the POMA and POMAShiny methods offered are also provided in the POMA package vignettes.

However, we agree with the reviewer that the app would benefit from more help materials and tutorials. For this reason, one of the future steps to be carried out in POMAShiny is the recording of different video tutorials that will be uploaded to a YouTube channel and users will also be able to access them from the application.

However, due to the time it will take, we believe we can benefit from user feedback when the app has been extended a bit more, in order to focus these

tutorials on the questions most frequently asked by users, as well as explaining new methods provided in later versions.

13) Statistical analysis: the authors should include limma in the *Univariate Analysis* section as linear model approaches are also univariate statistical methods.

We agree that limma is a univariate method and should be treated as such. We thank the reviewer for pointing this out. Following the reviewer's suggestion, we have included the limma method in the "Univariate analysis" panel in POMAShiny. Moreover, we have changed the position where limma is explained in the manuscript and have placed it just below where the univariate methods are explained (line 226).

14) The authors should detail if the linear model-based differential abundance analysis (e.g. by limma) allows also to adjust for confounding factors. Modeling e.g. batch effects or sex-age dependencies would be crucial for many data analyses.

We thank the reviewer for pointing this out. At the moment, POMAShiny and POMA allow users to adjust the ANCOVA and limma linear models for different confounding factors such as batch effects, age, sex or BMI. This functionality is one of the novel points of POMAShiny compared to other tools and it is extremely easy to use as users only need to include their covariates in the target file and access the tabs of the ANOVA and limma panels enabled specifically for this purpose.

However, thanks to the reviewer's comment, we realized that this issue was not explained clearly enough in the manuscript. Therefore, in response to this comment it has now been explained both for ANCOVA (lines 219–220) and limma (lines 234–238).

15) Avoid the use of modulable.

In line with the reviewer's proposal, "modulable" has been replaced by "customizable" in the manuscript.

16) line 22: web -> web-based

Changed according to the reviewer's proposal (line 27).

17) line 22-27: split the sentence. too complicated.

We agree with the reviewer. This has been changed according to the reviewer's suggestion.

18) line 45: the most readable -> as readable as possible

Changed according to the reviewer's comment on line 55.

19) line 119: what do the authors mean with "different modulable parameters".

We thank the reviewer for pointing this out. In response to the reviewer's comment and as mentioned above, "modulable" has been replaced by "customizable" in the manuscript.

20) line 120-122: reformulate this sentence. it is not clear what "by default but modulable" means. If euclidian distance is the default similarity measure but other options are available too, list these.

We agree that this needed to be reformulated. This statement has been clarified between lines 155 and 161. The default method for outlier detection uses Euclidean distances, however other distances, such as maximum, manhattan, canberra, and minkowski distances, are also provided.

21) line 135: what do the authors mean with "modulable plots"? customizable plots?

In accordance with the reviewer's comment and as mentioned above, "modulable" has been replaced by "customizable" in the manuscript.

22) line 141-143: split this sentence into two.

This has been done between lines 178 and 180.

23) line 144: what do the authors mean with a "classical heatmap".

We thank the reviewer for pointing this out. We have replaced "classical heatmap" with "clustered heatmap" (line 181).

Reviewer 2:

We thank the reviewer for his thoughtful review of our work, constructive comments and kind words. We have reviewed the manuscript thoroughly and addressed any errors or comments we came across with the help of the reviewers.

24) How would you compare the proposed set of web tool to other existing online resources? And What would distinguish it differently, and what would be its key advantages? Any limitation in using POMA or resources required?

We agree that a comparative section is mandatory and thank the reviewer for pointing this out. As the reviewer suggests, a section called "*Comparison with existing tools*" has been added to the manuscript (lines 363–406). This new section is focused on a comparison of the POMAShiny, W4M and MetaboAnalyst tools.

In addition to this new section, a detailed table comparing the aforementioned tools has been added to the manuscript for a faster, more understandable and more visual comparison (see Table 2).

The main points explained in the comparison section are:

- The interactive visualization and exploratory data analysis
- Preprocessing options (especially the outlier treatment)
- The ability to include covariates in the analysis and the statistical methods provided
- The ability to create random test and train sets in the predictive modelling strategies offered

25) Page 3 "To face this problem", better to use "to address this problem."

We thank the reviewer for pointing this out. This has been changed in line with the reviewer's comment (line 105).

26) Check code of conducts and remove/edit "[INSERT CONTACT METHOD]"

We thank the reviewer for this detailed comment. We overlooked this point. In response to the reviewer's comment, we have replaced it with "*polcaes@gmail.com".*

27) Regarding replacing a missing value with Zero. As you well know, there are many reasons for missing values, e.g., below LOQ or left-censored missing. If you use a determined value or zero, it may lead to certain biases, e.g., distortions of the distribution of missing variables and underestimations of the standard deviation. Therefore, I suggest making the caveats clear, and further explanations reference given to help guide the user to a better method choice, if possible.

We agree that this section was vague and needed to be clarified in the manuscript as well as focusing the text on what POMAShiny offered instead of focusing on the importance of imputation. We thank the reviewer for pointing this out.

For this reason, we have added a whole paragraph detailing the imputation options offered by POMAShiny (lines 107–126). In addition, as the reviewer suggests, we have clarified in the manuscript the different scenarios and recommendations regarding replacing missing values with zeros (lines 111–119). This section now reads:

"POMAShiny workflow offers a missing value imputation panel composed of different operations divided into three sequential steps: 1) distinction between zeros and missing values, 2) removal of features with a high percentage of missing values, and 3) imputation of remaining missing values.

First, if the data contain zeros or a combination of zeros and missing values, users can distinguish between them by using the algorithm provided by POMAShiny. For example, if the data contain both endogenous and exogenous compounds, the exogenous ones could be a real zero if they are not in the sample (absence), while the endogenous ones are unlikely to be real zeros (since they should always be in the sample, at least in very low concentrations). In that case, users could consider zeros as real zeros and impute only the missing values in the data. However, if users do not know the exact nature of the zeros in the data, or the difference between zeros and missing values, the authors recommend considering all zeros as missing values.

Second, users are able to remove features of the data with more than a specific percentage of missing values in all the study groups (20% allowed by default). Finally, several imputation methods for dealing with the remaining missing values after the two previous steps are provided in POMAShiny. The available methods are the imputation by zero, the half minimum imputation, the imputation by median, the imputation by mean, the imputation by minimum and the k-nearest neighbours imputation (where missing values are imputed using the k-nearest neighbours algorithm)."

28) In the Missing value section, are you considering "missingness" across entire data? If so, for example, if a sample has missing values beyond a certain percentage, would it be possible to remove that sample from the analysis?

We agree that this is an essential step to consider in metabolomics and proteomics studies and thank the reviewer for pointing this out.

Currently, this function has not yet been implemented in POMAShiny (except to remove features with a high percentage of missing values, not samples). However, it is planned that this useful function will be implemented in the next POMA and POMAShiny versions. Users will then be able to select two cut-off percentages, one for features and another for samples with a high percentage of missing values.

At present, while this methodology has not yet been implemented, users can manually remove those samples with a high percentage of missing values before uploading the data to POMAShiny or use the provided outlier detection/removal feature. For instance, if a sample contains a high percentage of missing values and these values are imputed by zero, this sample will most likely be categorized as an outlier with respect to its group (otherwise users could adjust the sensitivity to outliers). Therefore, this sample will be removed in the outlier detection panel and excluded from statistical analysis.

29) Any plans for blanks contaminants subtraction and dealing with "data cleaning." as part of pre-processing

We thank the reviewer for pointing this out. Blank subtraction is a very important step as it can include high bias in many metabolomics/proteomics studies. However, this operation is part of spectra processing in the early stages of the MS data analysis workflow. At the moment, POMAShiny does not address many of the steps prior to statistical analysis in MS workflows. One of the main reasons why these methods had not been included in POMAShiny is the computational cost of some of these procedures, which might hinder the portability of the Shiny application in certain systems. On the other hand, there are already several tools specialized in these purposes, both web tools (XCMS, Metaboanalyst, W4M, etc.) and R/Bioconductor packages (MSnbase, Spectra, mzR, etc.).

Therefore, although, as the reviewer points out, these methodologies could lead to an improvement of the tool, at the moment the incorporation of this methodology is not on the roadmap of the POMAShiny tool since there are already powerful tools for this purpose.

However, the authors are completely open to the requirements and needs of users, so if in the future these steps of the analysis are sufficiently required by users, of course we could consider including them in the application.

30) I would suggest differentiating between normalisation and scaling. In comparison, normalisation deals with the in samples variation, while scaling handles variations across variables (i.e. metabolites).

We agree that this point should be clarified in order not to confuse users and we thank the reviewer for pointing this out. In POMAShiny, the “normalization” process comprises both data transformation and scaling. However, this information was missing in the manuscript. Thanks to the reviewer’s comment, we were able to clarify this issue in the manuscript between lines 136 and 139.

31) The quality of the figures needs improvement for publication.

We thank the reviewer for pointing this out. In accordance with the reviewer’s request, the quality and resolution of the figures (especially those that are screenshots) have been improved. In addition, some minor changes have also been made to the figure notes, both in terms of the English and some small corrections and clarifications (below lines 77 and 298).

32) Finally, the help and online documentation can benefit from grammar and spelling revision to improve quality.

We agree that the help and online documentation would benefit from a revision of the grammar and spelling and thank the reviewer for pointing this out. The written English has been thoroughly reviewed and improved throughout the manuscript and the online documentation.
